# The increasing incidence and high body mass index-related burden of gallbladder and biliary diseases–A results from global burden of disease study 2019

**DOI:** 10.3389/fmed.2022.1002325

**Published:** 2022-12-02

**Authors:** Shuhua Liu, Maolin Yi, Juanjuan Qin, Fang Lei, Lijin Lin, Yi Li, Ming Zhuo, Weifang Liu, Xuewei Huang, Jingjing Cai, Xiaojing Zhang, Peng Zhang, Yanxiao Ji, Junming Ye, Hongliang Li

**Affiliations:** ^1^Department of Anesthesiology, First Affiliated Hospital of Gannan Medical University, Ganzhou, China; ^2^Department of Mammary Gland and Thyroid Gland, Huanggang Central Hospital of Yangtze University, Huanggang, China; ^3^Department of Cardiology, Renmin Hospital of Wuhan University, Wuhan, China; ^4^Institute of Model Animal, Wuhan University, Wuhan, China; ^5^School of Basic Medical Science, Wuhan University, Wuhan, China; ^6^Department of Cardiology, The Third Xiangya Hospital, Central South University, Changsha, China; ^7^Medical Science Research Center, Zhongnan Hospital of Wuhan University, Wuhan, China

**Keywords:** gallbladder and biliary diseases, incidence, high body mass index, years lived with disability, people aged 25–49 years

## Abstract

**Background:**

Gallbladder and biliary diseases are common gastrointestinal conditions associated with huge socioeconomic costs and are considered risk factors for cardiovascular diseases and digestive system cancers. The prevalence and incidence of gallbladder and biliary diseases have not received enough attention from 1990 to 2019. Several non-communicable diseases were associated with the incidence of gallbladder and biliary diseases. It is necessary to clarify the change in the incidence and disability burden of gallbladder and biliary diseases worldwide.

**Methods:**

Data on high body mass index (BMI)-related disease burden and incidence, years of life lost prematurely, and years lived with disability (YLDs) due to gallbladder and biliary diseases were obtained from the Global Burden of Disease 2019. The estimated annual percentage change was calculated to qualify the gallbladder and biliary disease burden change.

**Results:**

The global age-standardized incidence rate has increased from 585.35 per 100,000 (95% UI: 506.05–679.86) in 1990 to 634.32 per 100,000 (95% UI: 540.21–742.93) in 2019. And the increase in incidence was positively correlated with rising high BMI-related summary exposure value. The high BMI-related YLDs of gallbladder and biliary diseases have increased worldwide over time. Globally, the 25–49 age group suffered a rapid rise in incidence and high BMI attributable to the YLDs rate of gallbladder and biliary diseases.

**Conclusion:**

The global incidence and high BMI-related YLDs of gallbladder and biliary diseases remain prominent to increase over the past 30 years. Notably, the incidence and high BMI-related YLDs among people aged 25–49 years have rapidly increased over time. Therefore, high BMI should be emphasized in strategic priorities for controlling gallbladder and biliary diseases.

## Introduction

Gallbladder and biliary diseases (GBDs) is the most common gastrointestinal disease worldwide (1). Major complications from GBDs may lead to an increased risk of cancer, severe sepsis, and death (1–6). Moreover, it has been reported that GBDs increases the risk of cardiovascular death (7, 8). Therefore, the effective prevention of GBDs can result in a favorable effect in reducing the burden of non-communicable diseases (NCDs). Although it has been reported that the age-standardized prevalence, death, and disability-adjusted life years (DALYs) of GBDs declined from 1990 to 2019, the prevalence and incidence of GBDs have not received enough attention, especially in higher-income countries and younger populations (9). The uncontrolled incidence of GBDs may result in an escalated increase in death and DALYs burden in further years.

Among many risk factors of GBDs, obesity has increased dramatically. In 2015, excess body weight was estimated to affect 2 billion individuals and contributed to approximately 4 million deaths worldwide (10, 11). Obesity added a non-negatable burden to GBDs worldwide (9). The deaths and DALYs related to high body mass index (BMI) have increased from 24,000 (95% UI: 15–35) to 34,000 (95% UI: 22–49), and 453,000 (95% UI: 282–653) to 611,000 (95% UI: 391–866) between 2007 and 2017, respectively (12). Because of the high-quality health data of high-income countries, health problems in these regions are emerging more clearly (13–15). Data analysis from Korea showed a rising gallbladder disease-related burden among the young generation, which was correlated with obesity-related factors (14). It is noteworthy that high BMI-attributable DALYs had been poorly controlled globally, and they were more remarkable in higher-income regions and people aged 25+ years (9, 16). Since the higher-income regions and these populations have higher and more prolonged exposure to high BMI, the disease burden should be evaluated in them. Therefore, we performed a systematic analysis of the global incidence and disability burden of GBDs, highlighting the incidence and high BMI-related GBDs burden in higher-income countries and people aged 25+ years, especially in the younger populations. The finding from this study could provide a basis for making health policies to control the GBDs and relevant NCDs burden in the following decades.

## Data and methods

### Data source

We obtained the annual DALYs, years lived with disability (YLDs), years of life lost (YLLs), incident numbers and age-standardized rates (ASRs), and the high BMI-related data of gallbladder and biliary diseases, and high BMI-related summary exposure value (SEV) from an online database, the Global Health Data Exchange (GHDx) query tool.^1^ It provides accessible epidemiological data on 369 diseases and injuries and 87 attributable risk factors, which were collected from 204 countries and territories by location in the past 3 decades (17). To analyze the age gap of related burden due to gallbladder and biliary diseases and high BMI-related SEV, we choose those five age groups: 0–9 years, 10–24 years, 25–49 years, 50–74 years, and 75 plus years brought by this database. Socio-demographic Index (SDI) is the geometric mean of 0–1 indices of total fertility rate in those under 25 years old, mean education for those aged 15 years or older, and lag-distributed income per capita. It is a composite indicator of socio-demographic development degree correlated with health outcomes (18). To analyze the association between SDI and disease burden, 204 countries and territories were categorized into five SDI regions: low, low-middle, middle, high-middle, and high SDI regions. Moreover, they are also divided into 21 regions based on geographic location (17).

### Definitions

Gallbladder and biliary diseases include gallstones, cholecystitis, and other gallbladder and biliary tract diseases (18). International Classification of Disease-10 codes for gallbladder and biliary diseases included K80-K80.81 and K81-K83.9. High BMI for adults aged 20 and older is defined as BMI greater than 20–25 kg/m^2^ (17). High BMI for children (ages 1–19) is defined as being overweight or obese according to the International Obesity Task Force standard (17, 19, 20; Supplementary Table 1).

### Estimation methods

The general estimation methods for the Global Burden of Disease (GBD) Study are available elsewhere (17, 18). Briefly, the incidence and prevalence were estimated by the extensive population-representative data sources verified by relevant scientific reports and literature, health system data, and population surveys (13). Epidemiologic state-transition disease modeling software, Bayesian meta-regression method DisMod-MR 2.1, and MR-BRT were applied to maintain the consistency in disease estimates and adjusted for study-level differences in measurement methods and case definitions (18). Mortality was calculated by the International Classification of Disease-coded cause of vital registration data and household mortality surveys. Related statistical methods include reclassifying non-specific or unspecified codes, noise reduction algorithms, and the Cause of Death Ensemble model (18). Those methods were used to ensure the comparability of mortality data sources. To produce smoothed time trends across 204 countries and territories by location-specific covariates. DALYs were the sum of YLDs and YLLs. YLLs were equal to deaths multiplied by standard life expectancy at each age, and YLDs were equal to prevalence multiplied by disability weights for mutually exclusive sequelae of diseases and injuries. Uncertainty intervals (UIs) were used to reflect measurement errors in the presence of missing data (17, 18).

Exposures, attributable DALYs, attributable YLLs, attributable YLDs, and attributable deaths were estimated for 87 risk factors in GBD 2019 (17). GBD collaborators calculated the disease burden attributed to risk factors as the following (12, 16, 17): First, they included risk-outcome pairs based on reliable studies and estimated the relative risks (RRs) and exposure in specific age-sex-location-year included in the study. Second, the theoretical minimum risk exposure level (TMREL) was determined as the minimum exposure level of risk observed in published trials or studies. Then, the specific cause population attributable fractions (PAFs) attributable to risk factors were calculated. Third, deaths, YLLs, YLDs, and DALYs attributable to risk factors and corresponding PAFs were calculated in the study. For example, we estimated the PAF of the specific cause-related DALYs attributed to a risk factor. It quantifies the proportion of DALYs due to the specific cause that would be prevented if the risk factor distribution had been set to an optimal level in the population. We calculated the PAF of the disease burden attributable to a continuous risk factor j by age-sex-location-year by using the following specified formula:

$$\begin{array}{c} P\,A\,F_{\text{joasgt}}=\frac{\int_{x=1}^{\text{u}}R\,R_{j\,o\,a\,s\,g}\,p_{j\,a\,s\,g\,t}\,\left(x\right)\,dx-R\,R_{j\,o\,a\,s\,g}\,\left(T\,M\,R\,E\,L_{j\,a\,s}\right)}{\int_{x=l}^{u}R\,R_{j\,o\,a\,s\,g}\,\left(x\right)\,P_{j\,a\,s\,g\,t}\,\left(x\right)dx} \end{array}$$

where PAFjoasgt is the risk factor j-related PAF of cause o, for age group a, sex s, location g, and year t; RRjoasg(x) means the RR as a function of exposure level x for risk factor j, for cause o, age group a, sex s, and location g with the lowest exposure level at l and the highest at u; Pjasgt(x) is the distribution of exposure level x due to risk factor j, for age group a, sex s, location g, and year t; and TMREL_*jas*_ is the TMREL of risk factor j, for age group a, and sex s.

Then the disease burden attributable to high BMI is computed using the following equation:

$$A\,B_{\text{asgt}}=\sum_{o=1}^{w}D\,A\,L\,Y_{o\,a\,s\,g\,t}\,P\,A\,F_{o\,a\,s\,g\,t}$$

where ABasgt is the attributable disease burden for age group a, sex s, geography g, and year t; DALYoasgt is total DALYs for cause o (of w relevant outcomes) for age group a, sex s, geography g, and year t; PAFoasgt is the population attributable fraction (PAF) for cause o, age group a, sex s, geography g, and year t. The proportion of attributable deaths can be analogously computed by sequentially substituting each metric in place of DALYs in the equation above.

GBD2019 estimated the exposure distributions for 87 risk factors by the SEV. The SEV is the relative risk (RR)-weighted prevalence of exposure. It is used to compare the distribution of excess risk times exposure level among the people who are at the maximum risk (17). The SEVs range from 0 to 100. When the value is 0, it means a minimum risk for everyone in the population, and 100 indicates the population is at the highest level of risk. A decrease in SEV suggests reducing exposure to a specific risk factor, and an increase in SEV shows rising exposure (12, 17).

We first estimate risk factor r and cause c for specific SEV by the following equation (12, 17):

$$S\,E\,V_{\text{rc}}=\frac{\frac{{}_{P\,A\,F_{\text{rc}}}}{1{-}_{P\,A\,F_{\text{rc}}}}}{R\,R_{\max}-1}$$

SEV_*rc*_ is a specific risk factor r and outcome c pair. RRmax is the RR at the maximum exposure globally. PAF is the YLLs-related PAF due to risk factor r for cause c. For continuous risks, this is

$$R\,R_{\text{max}}=R\,R\,\frac{T\,M\,R\,E\,L-1^{s\,t}\,\exp o\,s\,u\,r\,e}{R\,R_{s\,c\,a\,l\,a\,r}}$$

$$\mathrm{if}\,\mathrm{protective},\mathrm{or}=R\,R\,\frac{99^{\text{th}}\,\exp o\,s\,u\,r\,e-T\,M\,R\,E\,L}{R\,R_{s\,c\,a\,l\,a\,r}}$$

When we obtained a set of risk-cause specific SEVs at the most-detailed risk, cause, age, sex, and location for all years, we averaged across causes to produce the final risk-specific SEV_*r*_:

$$S\,E\,V_{\text{r}}=\frac{1}{N\,\left(c\right)\,c}\,\sum_{c}S\,E\,V_{r\,c}$$

Where N(c) is the total number of outcomes c for a specific risk factor r.

### Statistical analysis

We applied the incidence, YLLs, and YLDs rate for gallbladder and biliary diseases and high BMI-related SEV, then calculated the corresponding estimated annual percentage change (EAPC) with 95% confidence intervals (CI) to assess the change in gallbladder and biliary disease burden and high BMI-related exposure from 1990 to 2019.

Our study used the age-standardized rate (ASR) and EAPC to quantify the disease burden trends from 1990 to 2019. The ASR is the rate after excluding the effects of age. It was calculated based on the following formula (21):

$$\mathrm{Age}-\mathrm{standardized}\,\mathrm{rate}=\frac{\sum_{\text{i}=1}^{A}a\,i\,w\,i}{\sum_{i=1}^{A}w\,i}\times 100,000$$

Where ai is the ith age group and the number of persons (or weight) (wi) in the same age subgroup i of the chosen reference standard population. Then, the value was divided by the sum of standard population weights.

Estimated annual percentage change is a summary and commonly used measure of the ASR trend in the specific study period. Firstly we assumed that the natural logarithm of ASR is linear over time. So Y = α + βX + ε, Y = ln(ASR), X = calendar year, and ε = the error term. EAPC = [exp(β)−1], and the corresponding 95% CI were obtained from the linear regression model (21). If the EAPC and the lower boundary of its 95% CI were both positive, the ASR was considered to rise. Instead, if the EAPC estimation and the upper boundary were negative, the ASR was considered to drop (21). Moreover, we analyzed the correlation between high BMI-related summary exposure value and the age-standardized incidence, YLDs, and YLLs rate to SDI by the Pearson correlation analysis, *p* < 0.05 regarded as statistically significant.

## Results

### The global incidence of gallbladder and biliary diseases increased from 1990 to 2019

**FIGURE 1:**
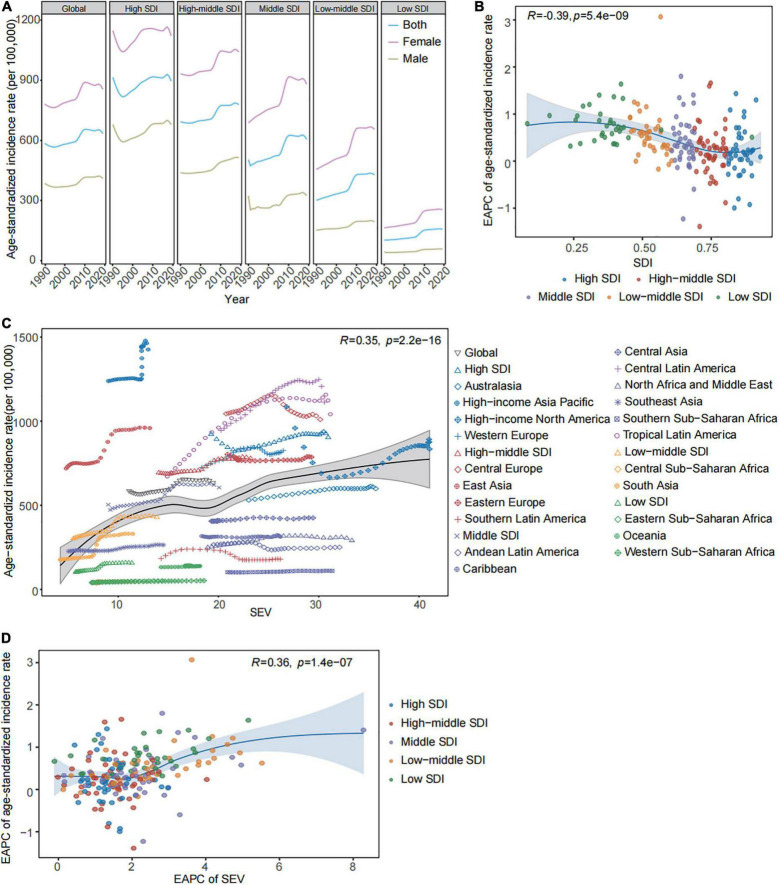
The gallbladder and biliary diseases incidence and high BMI-related SEV in global and in regions from 1990 to 2019. **(A)** The trend in the age-standardized incidence rates of gallbladder and biliary diseases by sex across global and different SDI regions from 1990 to 2019. **(B)** The correlations between the EAPC of age-standardized incidence rates and SDIs across 204 countries and territories. **(C)** The correlations between age-standardized incidence rates and high BMI-related SEVs in global and different regions. **(D)** The correlations between the corresponding EAPC in high BMI-related SEVs and the age-standardized incidence rates across 204 countries and territories. BMI, body mass index; SDI, socio-demographic index; EAPC, estimated annual percentage change; SEV, summary exposure value.

The global incidence cases of gallbladder and biliary diseases dramatically increased from 26.35 million (95% UI: 22.79–30.60) in 1990 to 52.00 million (95% UI: 44.20–61.21) in 2019 (EAPC = 2.70 [95% CI: 2.58–2.81]). The age-standardized incidence rate (ASIR) increased from 585.35 per 100,000 (95% UI: 506.05–679.86) in 1990 to 634.32 per 100,000 (95% UI: 540.21–742.93) in 2019 (EAPC = 0.59 [95% CI: 0.48–0.69]) (Table 1). The incidence cases of gallbladder and biliary diseases presented substantial increases across all SDI regions since 1990 (Table 1). In the high SDI region, the ASIR due to gallbladder and biliary diseases was reduced from 1990 to 1993, and whereafter, an increase appeared from 1994 to 2019. There were uptrends in ASIRs at high-middle SDI (EAPC = 0.58 [95% CI: 0.49–0.66]), middle SDI (EAPC = 1.12 [95% CI: 0.96–1.27]), low-middle SDI (EAPC = 1.53 [95% CI: 1.36–1.71]), low SDI areas (EAPC = 1.92 [95% CI: 1.68–2.15]) during the past 30 years (Table 1 and Figure 1A). The ASIRs increased with rising SDIs, but the corresponding EAPC was negatively correlated with SDIs (*R* = −0.39, *p* < 0.05) (Figures 1A,B and Table 1). We have illustrated the influence of high BMI-related age-standardized SEV on the ASIR. Figures 1C,D showed a positive correlation between ASIRs and high BMI-related age-standardized SEVs (*R* = 0.35, *p* < 0.05). Meanwhile, a positive correlation has also existed in the corresponding EAPC (*R* = 0.36, *p* < 0.05) in the study. The rising trends of age-standardized SEVs due to high BMI appeared in global and all SDI regions over time (Supplementary Figure 1A and Supplementary Table 2). The high BMI-related age-standardized SEV increased with rising SDI, and the corresponding EAPC was negatively correlated with SDIs (*R* = −0.34, *p* < 0.05) (Supplementary Figures 1A,B).

**TABLE 1:** The gallbladder and biliary disease burden in global and in different SDI regions from 1990 to 2019.

Location	Case ×10^3^ (95% UI) in 1990	Case ×10^3^ (95% UI) in 2019	EAPC of case (95% CI)	ASR per 100,000 (95% UI) in 1990	ASR per 100,000 (95% UI) in 2019	EAPC of ASR (95% CI)
**Incidence**						
Global	26351.36 (22790.17, 30598.25)	52003.77 (44202.14, 61211.62)	2.70 (2.58, 2.81)	585.35 (506.05, 679.86)	634.32 (540.21, 742.93)	0.59 (0.48, 0.69)
High SDI	8735.62 (7487.76, 10223.57)	12860.58 (11042.25, 15000.36)	1.78 (1.65, 1.90)	922.35 (791.33, 1078.94)	903.10 (775.89, 1051.05)	0.35 (0.22, 0.47)
High-middle SDI	7819.00 (6730.09, 9100.64)	14445.16 (12091.79, 17193.07)	2.34 (2.25, 2.43)	693.76 (596.75, 805.64)	779.29 (656.39, 919.74)	0.58 (0.49, 0.66)
Middle SDI	6849.51 (5876.46, 8003.93)	16564.48 (13930.96, 19595.42)	3.38 (3.23, 3.53)	503.59 (433.29, 584.82)	634.27 (538.90, 741.34)	1.12 (0.96, 1.27)
Low-middle SDI	2549.29 (2191.92, 2973.53)	6865.09 (5883.53, 8041.69)	3.81 (3.63, 3.99)	300.92 (262.00, 349.18)	428.16 (368.58, 498.21)	1.53 (1.36, 1.71)
Low SDI	389.76 (333.27, 454.64)	1253.65 (1062.79, 1455.53)	4.51 (4.28, 4.74)	103.70 (90.24, 120.36)	159.28 (135.84, 184.47)	1.92 (1.68, 2.15)
**YLDs**						
Global	2685.06 (1721.29, 3913.05)	4061.84 (2595.49, 5953.10)	1.54 (1.44, 1.63)	59.71 (38.41, 86.83)	49.33 (31.51, 71.96)	−0.58 (−0.68, −0.49)
High SDI	604.00 (389.84, 880.90)	797.41 (519.40, 1163.42)	0.99 (0.95, 1.03)	63.61 (40.98, 92.97)	56.53 (36.44, 81.79)	−0.39 (−0.43, −0.35)
High-middle SDI	798.61 (513.11, 1157.97)	1044.16 (667.26, 1533.33)	0.93 (0.84, 1.01)	70.94 (45.35, 102.40)	55.90 (35.61, 82.18)	−0.86 (−0.95, −0.76)
Middle SDI	839.93 (534.84, 1238.61)	1360.20 (858.23, 2012.10)	3.13 (2.92, 3.34)	63.65 (40.77, 92.75)	51.52 (32.61, 75.80)	−0.61 (−0.76, −0.46)
Low-middle SDI	377.07 (240.26, 550.98)	711.28 (454.78, 1033.74)	2.39 (2.22, 2.56)	46.74 (29.98, 67.99)	44.65 (28.64, 64.74)	0.00 (−0.16, 0.16)
Low SDI	64.52 (41.53, 94.81)	147.36 (93.75, 214.88)	3.13 (2.92, 3.34)	18.37 (11.76, 26.77)	19.30 (12.33, 27.91)	0.43 (0.23, 0.63)
**YLLs**						
Global	1919.76 (1483.62, 2121.45)	2290.90 (1970.33, 2524.61)	0.59 (0.42, 0.75)	47.05 (37.04, 51.88)	28.92 (24.89, 31.76)	−1.68 (−1.80, −1.57)
High SDI	292.41 (257.10, 322.51)	398.18 (333.55, 444.03)	1.31 (1.08, 1.54)	28.20 (24.82, 30.99)	19.98 (17.12, 22.23)	−0.98 (−1.17, −0.80)
High-middle SDI	471.82 (366.18, 514.23)	436.20 (388.41, 496.94)	−0.49 (−0.66, −0.32)	45.67 (35.63, 49.67)	22.27 (19.83, 25.14)	−2.65 (−2.76, −2.54)
Middle SDI	603.64 (448.50, 670.65)	698.12 (592.41, 799.71)	1.81 (1.73, 1.88)	55.25 (42.38, 61.50)	29.69 (25.36, 33.84)	−2.20 (−2.30, −2.09)
Low-middle SDI	349.67 (250.99, 418.57)	425.01 (365.80, 488.17)	0.70 (0.51, 0.90)	49.53 (36.06, 59.39)	30.40 (26.51, 35.06)	−1.66 (−1.78, −1.54)
Low SDI	201.07 (134.36, 257.36)	331.78 (246.26, 423.83)	1.81 (1.73, 1.88)	65.27 (49.71, 83.05)	52.85 (40.67, 70.35)	−0.68 (−0.71, −0.66)

ASR, age-standardized rate; SDI, socio-demographic index; YLDs, years lived with disability; YLLs, years of life lost; UI, uncertainty intervals; CI, confidence intervals; EAPC, estimated annual percentage change.

The uptrends in ASIRs appeared in 15 of 21 GBD regions over time. In 2019, the highest ASIR was observed at high-income Asia Pacific (1,426.23 [95% UI: 1,211.62–1,670.16] per 100,000), Central Latin America (1,112.70 [95% UI: 958.42–1,295.35] per 100,000) (Supplementary Table 3). The increases in ASIRs were found in 163 of 204 countries and territories during the study period. Gallbladder and biliary diseases have the highest incidence in Italy (1,718.36 [95% UI: 1,441.49–2,039.23] per 100,000), Japan (1,614.18 [95% UI: 1,366.14–1,898.78] per 100,000), and the United Kingdom (1,593.45 [95% UI: 1,339.08–1,878.10] per 100,000) in 2019 (Supplementary Table 4).

### The years lived with disability as the main component of disability-adjusted life years were poorly controlled relative to years of life lost prematurely across global and most socio-demographic index regions

**FIGURE 2:**
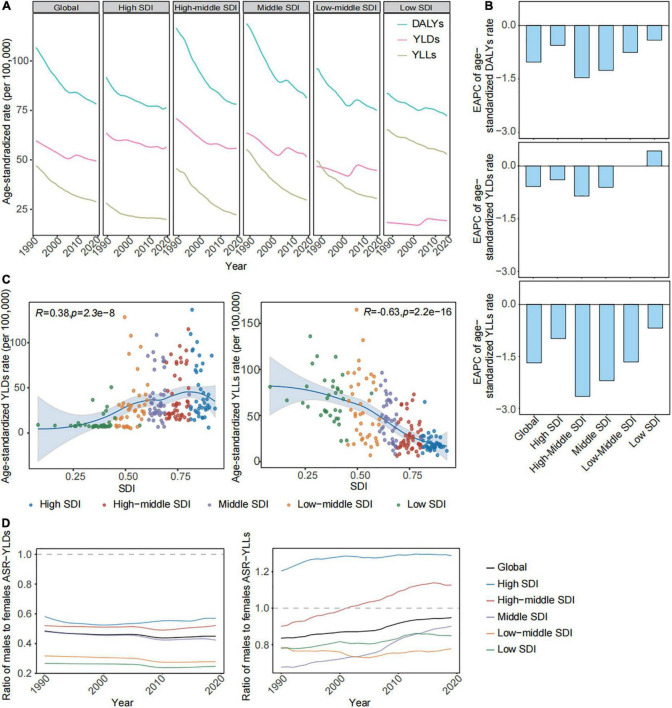
The age-standardized YLLs, YLDs, and DALYs rates of gallbladder and biliary diseases in global and in regions from 1990 to 2019. **(A)** The trend in the age-standardized YLLs, YLDs, and DALYs rates of gallbladder and biliary diseases across global and different SDI regions from 1990 to 2019. **(B)** The EAPC in the age-standardized YLLs, YLDs, and DALYs rates of gallbladder and biliary diseases across the global and different SDI regions from 1990 to 2019. **(C)** The correlations of the age-standardized YLLs and YLDs rate with the SDIs across 204 countries and territories. **(D)** The ratio of males to females in the age-standardized YLDs and YLLs rates of gallbladder and biliary diseases in global and in different SDI regions from 1990 to 2019. SDI, socio-demographic index; DALYs, disability adjusted of life years; YLDs, years lived with disability; YLLs, years of life lost; EAPC, estimated annual percentage change.

The age-standardized DALYs rates have decreased, but the trends in the two components of DALYs were not corresponding across global and different SDI regions in the interval. YLDs was the main proportion of DALYs due to gallbladder and biliary diseases across global and low-middle to high areas, but the low area has the opposite expression (Figure 2A). The downtrends of age-standardized YLDs rates were only observed in high SDI (EAPC = −0.39 [95% CI: −0.43 to −0.35]), high-middle SDI [EAPC = −0.86 (95% CI: −0.95 to −0.76)], and middle SDI areas (EAPC = −0.61 [95% CI: −0.76 to −0.46]) during the past 30 years. There was a non-significant change in the age-standardized YLDs rate in the low-middle SDI region (EAPC = 0.00 [95% CI: −0.16 to 0.16]), and even a slight rise was found in the low SDI region at the time (EAPC = 0.43 [95% CI: 0.23–0.63]). The age-standardized YLLs rates due to gallbladder and biliary diseases have rapidly dropped throughout all SDI regions over time (Table 1 and Figure 2B). Figure 2C showed that the age-standardized YLDs rates (*R* = 0.38, *p* < 0.05) were positively related to SDIs. The age-standardized YLDs rates across higher and middle SDI areas exceeded those with lower SDI (Figure 2A). But the age-standardized YLL rates (*R* = −0.63, *p* < 0.05) were negatively correlated with SDIs, the highest age-standardized YLLs rate and the corresponding slowest decline was observed in low SDI region (EAPC = −0.68 [95% CI: −0.71 to −0.66]) (Figures 2A–C and Table 1). The sharp declines in the age-standardized YLLs rates mainly led to the decreasing age-standardized DALYs rates in global and most SDI regions during the study. In 2019, there were slight differences in age-standardized DALYs rates because of the different changes in age-standardized YLLs and YLDs rates across different SDI regions.

The ASRs of YLLs and YLDs have declined in the majority of GBD regions, countries, and territories over time (Supplementary Table 3). In 2019, age-standardized YLDs rates attributable to gallbladder and biliary diseases were the most frequent in Central Latin America (100.62 [95% UI: 64.01–149.28] per 100,000), United Kingdom (136.51 [95% UI: 86.40–199.28] per 100,000), Honduras (128.37 [95% UI: 82.34–192.13] per 100,000), and Italy (115.01 [95% UI: 72.88–168.92] per 100,000). The greatest age-standardized YLL rates related to gallbladder and biliary diseases were found in Eastern Sub-Saharan Africa (88.41 [95% UI: 62.06–143.80] per 100,000) and Honduras (164.76 [95% UI: 116.57–227.61] per 100,000) (Supplementary Tables 3, 4).

### The age-standardized years lived with disability rate due to high body mass index has escalated from 1990 to 2019 worldwide

As shown in Table 2, the global high BMI-attributed age-standardized YLDs rate increased from 13.97 per 100,000 (95% UI: 6.48,26.43) to 16.10 per 100,000 (95% UI: 8.34–28.28) over the past 30 years (EAPC = 0.60 [95% CI: 0.54–0.66]). The high BMI-related age-standardized YLLs rate reduced to 8.84 per 100,000 (95% UI: 5.51–12.79) in 2019 worldwide (EAPC = −0.22 [95% CI: −0.33 to −0.11]). The cases and age-standardized PAFs of YLDs and YLLs due to high BMI for GBDs have rapidly increased in global and different SDI areas in the interval. Moreover, the age-standardized YLDs rates from high BMI increased with SDIs and have increased throughout most SDI regions over time. The age-standardized YLLs rate attributed to high BMI also showed increasing trends in middle and lower SDI regions. Notably, the fastest rise in high BMI contributes to the age-standardized YLDs and YLLs rates of GBDs observed in lower SDI regions during the study.

**TABLE 2:** The gallbladder and biliary disease burden due to high body mass index in global and in different SDI regions from 1990 to 2019.

Location	Case ×10^3^ (95% UI) in 1990	Case ×10^3^ (95% UI) in 2019	EAPC of case (95% CI)	Age-standardized PAF (95% UI) in 1990	Age-standardized PAF (95% UI) in 2019	EAPC of age-standardized PAF (95% CI)	ASR per 100,000 (95% UI) in 1990	ASR per 100,000 (95% UI) in 2019	EAPC of ASR (95% CI)
**YLDs**									
Global	605.92 (278.62, 1144.08)	1337.89 (692.11, 2339.11)	2.91 (2.85, 2.97)	0.23 (0.13, 0.36)	0.33 (0.21, 0.46)	1.19 (1.15, 1.24)	13.97 (6.48, 26.43)	16.10 (8.34, 28.28)	0.60 (0.54, 0.66)
High SDI	198.42 (98.12, 353.08)	314.90 (169.26, 533.51)	1.69 (1.60, 1.79)	0.32 (0.20, 0.46)	0.39 (0.26, 0.53)	0.75 (0.68, 0.82)	20.54 (10.14, 36.39)	22.20 (12.01, 37.88)	0.35 (0.28, 0.43)
High-middle SDI	220.08 (107.17, 399.63)	371.47 (190.94, 642.46)	1.85 (1.77, 1.93)	0.28 (0.17, 0.41)	0.34 (0.22, 0.49)	0.77 (0.75, 0.78)	19.70 (9.59, 35.72)	19.25 (9.91, 33.59)	−0.09 (−0.19, 0.00)
Middle SDI	136.35 (53.66, 283.48)	437.86 (220.64, 775.78)	4.32 (4.19, 4.44)	0.17 (0.08, 0.29)	0.31 (0.20, 0.45)	2.26 (2.17, 2.36)	10.62 (4.18, 22.09)	16.23 (8.18, 28.81)	1.65 (1.54, 1.75)
Low-middle SDI	44.21 (15.15, 98.51)	185.41 (91.39, 336.91)	5.31 (5.17, 5.44)	0.12 (0.05, 0.23)	0.26 (0.16, 0.39)	2.64 (2.57, 2.71)	5.85 (2.04, 13.07)	11.74 (5.79, 21.25)	2.64 (2.52, 2.75)
Low SDI	6.59 (2.29, 14.59)	27.71 (12.80, 51.57)	5.36 (5.10, 5.62)	0.12 (0.05, 0.22)	0.21 (0.12, 0.32)	2.02 (1.92, 2.11)	2.14 (0.75, 4.71)	4.00 (1.85, 7.41)	2.46 (2.25, 2.66)
**YLLs**									
Global	369.89 (198.06, 596.23)	709.55 (442.97, 1025.44)	2.34 (2.19, 2.49)	0.20 (0.11, 0.32)	0.31 (0.20, 0.43)	1.48 (1.44, 1.53)	9.61 (5.20, 15.36)	8.84 (5.51, 12.79)	−0.22 (−0.33, −0.11)
High SDI	90.38 (51.87, 133.83)	143.37 (87.57, 206.90)	1.84 (1.67, 2.02)	0.31 (0.19, 0.45)	0.37 (0.24, 0.51)	0.64 (0.59, 0.70)	8.69 (4.99, 12.86)	7.40 (4.60, 10.57)	−0.35 (−0.49, −0.21)
High-middle SDI	130.24 (76.04, 197.26)	169.56 (108.57, 239.88)	0.77 (0.62, 0.93)	0.28 (0.17, 0.41)	0.38 (0.25, 0.52)	1.22 (1.17, 1.27)	12.63 (7.36, 19.03)	8.55 (5.46, 12.11)	−1.45 (−1.55, −1.35)
Middle SDI	97.40 (47.92, 167.30)	230.33 (143.92, 330.70)	3.11 (3.02, 3.20)	0.16 (0.08, 0.28)	0.32 (0.21, 0.45)	2.49 (2.41, 2.56)	9.07 (4.36, 15.72)	9.46 (5.88, 13.70)	0.24 (0.17, 0.32)
Low-middle SDI	34.37 (14.06, 66.83)	108.53 (64.86, 163.54)	4.25 (4.12, 4.38)	0.11 (0.05, 0.21)	0.26 (0.16, 0.37)	3.11 (3.02, 3.21)	5.47 (2.20, 10.59)	7.79 (4.60, 11.74)	1.41 (1.33, 1.49)
Low SDI	17.25 (6.37, 34.94)	57.25 (29.91, 97.69)	4.37 (4.15, 4.58)	0.10 (0.04, 0.20)	0.19 (0.10, 0.31)	2.28 (2.16, 2.39)	6.83 (2.56, 13.72)	10.27 (5.27, 17.78)	1.58 (1.45, 1.71)

ASR, age-standardized rate; SDI, socio-demographic index; YLDs, years lived with disability; YLLs, years of life lost; UI, uncertainty intervals; CI, confidence intervals; EAPC, estimated annual percentage change; PAF, population attributable fraction.

High BMI contributed to the age-standardized YLDs rates having increased among 13 of 21 GBD regions, and the age-standardized YLLs rates having uptrends among 9 of 21 GBD regions in this time interval (Supplementary Table 5). In 2019, the age-standardized YLDs rate due to high BMI was the highest in Central Latin America (45.04 [95% UI: 24.25–75.03] per 100,000) among 21 GBD regions and the United Kingdom (62.39 [95% UI: 33.85–105.02] per 100,000), Mexico (51.39 [95% UI: 27.76–84.72] per 100,000) across 204 countries and territories. The greatest age-standardized YLLs rate related to high BMI was in Tropical Latin America (27.24 [95% UI: 18.21–37.16] per 100,000) among 21 GBD regions and Honduras (60.39 [95% UI: 32.8–103.51] per 100,000) across 204 countries and territories (Supplementary Tables 5, 6).

### There was a rapid increase in the incidence rate and high body mass index-related years lived with disability rate across the 25–49 age group worldwide over time

For the subgroup analysis of genders in 2019, the ASIRs and age-standardized YLDs rates of gallbladder and biliary diseases among females were higher than among males globally and across all SDI regions (Figures 1A, 2D). The same-sex difference in age-standardized YLLs rates was found in global, middle, and lower SDI regions. But the age-standardized YLLs rates among males exceeded females in higher SDI regions (Figure 2D). Moreover, the age-standardized YLDs and YLLs rates due to high BMI of gallbladder and biliary diseases for females also exceeded males throughout global and all SDI regions (Supplementary Table 7).

**FIGURE 3:**
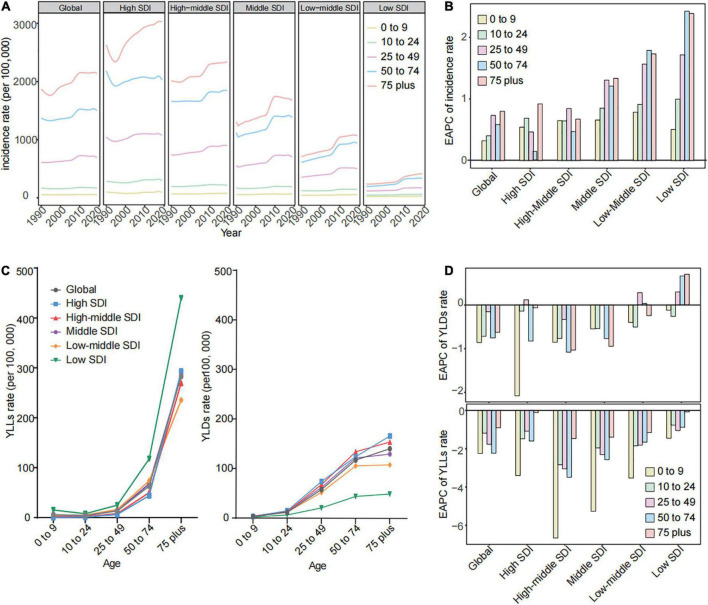
The gallbladder and biliary diseases incidences and high BMI-related SEVs by age in global and different SDI regions from 1990 to 2019. **(A)** The trend in incidence rates of gallbladder and biliary diseases by age across global and different SDI regions from 1990 to 2019. **(B)** The EAPC of incidence rates for gallbladder and biliary diseases by age across global and different SDI regions from 1990 to 2019. **(C)** The YLDs and YLLs rates of gallbladder and biliary diseases by age across global and different SDI regions in 2019. **(D)** The EAPC of YLDs and YLLs rates for gallbladder and biliary diseases by age across global and different SDI regions from 1990 to 2019. BMI, body mass index; SDI, socio-demographic index; EAPC, estimated annual percentage change; SEV, summary exposure value; YLDs, years lived with disability; YLLs, years of life lost.

The gallbladder and biliary diseases occur more in adults, and the incidence rates increased with age, peaking at ≥75 years old in global and all SDI regions over time (Figure 3A). The incidence rate of people aged ≥ 25 years old increased across global and all SDI regions, with the fastest increase in lower SDI areas (Figure 3B). The incidence rate for the 25–49 age group has a relatively sharper rise in global, high, and middle SDI regions, even becoming the fastest rise in the high-middle SDI region in this time interval (Figure 3B). Meanwhile, the high BMI-related SEVs have rapidly increased among all age brackets, and the fastest rise was found in the 25–49 age group across global and all SDI regions (Supplementary Figures 1C,D and Supplementary Table 2). Among the 25–49 age bracket, the YLDs rate exceeded the YLLs rate in global and different SDI regions (Figure 3C). Their YLDs rate significantly rose across high and low-middle SDI regions, with the minimum decline in global, high-middle, and middle SDI regions over time (Figure 3D). The YLLs focused on people aged 75 years onward; the YLLs rate among them has declined in the interval in global, high-middle to low-middle SDI regions (Figures 3C,D). Moreover, the high BMI-related SEVs have rapidly increased and risen with SDI among all age brackets, and the fastest rise appeared in the 25–49 age group across global and all SDI regions (Supplementary Figures 1C,D).

**FIGURE 4:**
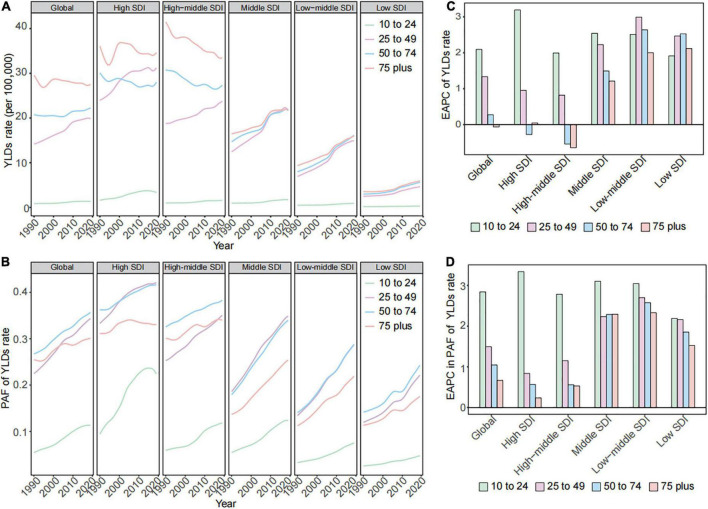
The changes in high BMI contributed to YLDs burden for gallbladder and biliary diseases by age in global and in different SDI regions from 1990 to 2019. **(A)** The trend in YLDs rates due to high BMI for gallbladder and biliary diseases by age in global and in different SDI regions from 1990 to 2019. **(B)** The EAPC of YLDs rates due to high BMI for gallbladder and biliary diseases by age in global and in different SDI regions from 1990 to 2019. **(C)** The trend in PAF of YLDs rates due to high BMI for gallbladder and biliary diseases by age in global and in different SDI regions from 1990 to 2019. **(D)** The EAPC in PAF of YLDs rates due to high BMI for gallbladder and biliary diseases by age in global and in different SDI regions from 1990 to 2019. BMI, body mass index; SDI, socio-demographic index; EAPC, estimated annual percentage change; PAF, population-attributable fraction; YLDs, years lived with disability.

The YLDs rate and corresponding PAF related to high BMI focused on people aged 25 years onward in global and all SDI regions (Figures 4A,B). The highest YLDs rate and corresponding PAF due to high BMI were observed in higher SDI regions, while the greatest uptrends were observed in middle and lower SDI regions. The 25–49 age group has the fastest rise in the YLDs rate and corresponding PAF due to high BMI among adults in global and most SDI regions over time (Figures 4A–D). ≥75 years age group was the primary age group for the YLLs rate due to high BMI across global and all SDI regions (Supplementary Figure 2A). The most prominent uptrend in YLLs rates attributed to high BMI shown in lower SDI regions over time (Supplementary Figure 2B). Although the YLLs rate due to high BMI among the 25–49 age group was low and had a mild change, the PAF of YLLs rate also has the fastest rise among adults (Supplementary Figures 2A–D).

## Discussion

This systematic evaluation shows up-to-date patterns of the worldwide distribution, temporal trends in incidence, and high BMI-related burden for gallbladder and biliary diseases. From 1990 to 2019, the incidences of gallbladder and biliary diseases have rapidly increased globally and in most regions. A positive association was found between the disease incidence and increased high BMI exposure. Notably, there was a rapidly rising incidence in people aged 25–49 years, and the YLDs of gallbladder and biliary diseases among them were poorly controlled worldwide. The YLDs due to high BMI and the exposure level of high BMI for them have most prominently increased among adult populations across global and most SDI regions in the study period.

A rapid rise in the incidence rates for gallbladder and biliary diseases was found throughout most regions from 1990 to 2019. The uptrend is supported by previous epidemiological studies of gallbladder and biliary diseases in local areas or countries. For instance, the incidence of primary sclerosing cholangitis increased by about 50% in the UK from 1991 to 2001 (22). The ASIR of gallstone-related pancreatitis had risen from 10.8 to 19.1 cases per 100,000 individuals between 1990–1994 and 2010–2013 in Sweden (23). Our analysis revealed the change in the incidence of gallbladder and biliary diseases was positively associated with the rise in high BMI-related exposure. Park et al. also verified that with an increment of 5 kg/m^2^ of BMI, the risk of non-cancer biliary tract diseases increased by 1.40 (24). Aune et al. conducted a dose-response meta-analysis of cohort studies of BMI and risk of gallbladder disease. They confirmed a positive association between both general and abdominal fatness and the risk of gallbladder disease (25). A Swedish twin study examined that overweight (BMI, 25–30 kg/m^2^) and obesity (BMI > 30 kg/m^2^) were prominently associated with the risk for symptomatic gallstone diseases in the twin population (26). Global age-standardized mean BMI increased from 21.7 to 24.2 kg/m^2^in men and from 22.1 to 24.4 kg/m^2^ in women from 1975 to 2014. The age-standardized prevalence of obesity increased from 3.2 to 10.8% in men and from 6.4 to 14.9% in women during the same period (27). Changes in the food environment and systems are likely to be major drivers of the rise in obesity prevalence or mean BMI. The fast-growing consumption of energy-dense foods and the efficient marketing of those foods could explain excess energy intake and weight gain across populations (28). The reduced physical activity caused by urbanization and others in the modernized environment was considered the potential driver for high BMI (20).

Moreover, We speculate the following changes might also partly account for the rising incidence rate of gallbladder and biliary diseases: (1) The introduction of high-calorie, carbohydrate, and fat diets and physical inactivity were related to the increased risk of gallbladder and biliary diseases (1, 29). As global nutrition transitioned, traditional diets were replaced by diets with high sugars, fats, oils, and meats (30–32). The worldwide prevalence of physical inactivity has increased from 17.4 to 31.1% among adults during the past decade (33, 34). (2) The highly prevalent metabolic syndrome, type 2 diabetes mellitus (T2DM) may be related to the high risk of gallbladder and biliary diseases (1). The global diabetes prevalence (T2DM accounts for approximately 90% of total diabetes) increased to 9.3% in 2019 and will continue to rise to 10.2% in 2030 (35). Yuan et al. verified that T2DM was one of the independent causal roles in gallstone disease (36). The prevalence of metabolic syndrome increased rapidly from 13.7 to 24.2% in China from 2001 to 2010–2012 (37, 38). In a population study from China, metabolic syndrome was prominently associated with gallbladder and biliary stones. There was a significant dose effect between the rising number of metabolic syndrome components and the risk of biliary tract stones. Dyslipidemia was associated with excess risks of biliary stones (39). However, the association between high blood pressure and gallbladder and biliary diseases was inconsistent in previous studies (39–41). (3) Aging population may be a driving factor for gallbladder and biliary disease incidence (1, 9, 42). (4) The widespread use of imagological examination and invasive manipulation may also lead to an increase in the detectable rate or incidence of gallbladder and biliary diseases (5, 43).

A higher incidence rate of gallbladder and biliary diseases was observed in higher SDI regions. In Asia, the prevalence of gallstone disease was reported to be 6.1% in Korea (high SDI country), 3.8–6.1% in China (high-middle SDI country), and 3.1% in India (middle SDI country) (44, 45). The distribution of incidence was parallel with the high BMI-related SEV among different SDI regions. In general, the obesity prevalence and mean BMI were higher in high-income countries than in low- and middle-income countries (LMICs) (20, 27). The prevalence of T2DM, physical inactivity, and consumption of ultra-processed products among high-income countries was higher than in those with low income may also stimulate this distribution of incidence among different SDI regions (32, 33). It reported that the diabetes prevalence is higher in high-income (10.4%) than in low-income countries (4.0%) (45). Moreover, this difference in incidence among SDI regions may be influenced by the high detection rates by the complete data coverage, vital record data, surveillance systems and data sets, efficient national health policies of NCDs, and related risk factors in high-income countries (13–15).

The faster rise of ASIR appeared in lower SDI regions. The increase in ASIR was also positively associated with the rising rates of high BMI-related SEV. It reported that a more prominent increase in mean BMI and obesity prevalence was observed at LMICs than in high-income countries (27). In addition, it may result from the rapid increase in diabetes, the aging population, and the introduction of high-calorie, carbohydrate, and fat diets in developing countries (46). There will be a 42% increase in diabetes prevalence in developed countries and a 170% increase in developing countries from 1995 to 2025 (47). The consumption of ultra-processed products is more rapidly increasing among middle-income countries than in high-income countries. A rapidly growing number of older people was observed globally, especially in developing countries. It is predicted that the number of older people will continuously rise to 2.1 billion in 2050, and 80% of them living in developing countries (48).

This study showed that the incidence and high BMI-related YLDs rate of gallbladder and biliary diseases increased rapidly in people aged 25–49 years worldwide over time. According to our results, the YLDs was poorly controlled because of the rapid increase in the high BMI-related YLDs rate among people aged 25–49 years. Wang et al. reported that the annual increase rate in the prevalence of overweight and obesity was highest in individuals aged 18–44 years among men (49). The epidemiologic study indicated that obesity prevalence has rapidly increased with age among adults and peaked at 50–64 years old (20). Data analysis from Korea showed a rising prevalence of gallbladder diseases among the younger generation, which was correlated with obesity-related factors (50). The significantly increased high BMI-related exposure may greatly stimulate the fast-growing incidence for those populations. Secondly, the dramatically rising prevalence of type 2 diabetes and metabolic syndrome in young people may partly account for the change in incidence. In China, there was an 88% increase in diabetes prevalence among the 35–44 age group from 1994 to 2000 (51). The incidence and prevalence of diabetes continued to increase at a greater rate for younger individuals than older from 1990 to 2012 in the US (52). From 2011 to 2016, metabolic syndrome prevalence increased significantly among the younger generation aged 20–39 years (from 16.2 to 21.3%; women, from 31.7 to 36.6%;men) in the US (53). Therefore, metabolic disorders may be the main driving factor in poorly controlled disease burden of gallbladder and biliary diseases among people aged 25–49. The increasing incidence of metabolic diseases in young people partially results from sedentary living and high-energy dietary intake (35). Fast foods, ultra-processed food, and takeaway food with high calories and fat content are more popular with the young generation. Increased sedentary lifestyles by the modernized work environment and lost time because of work commitments all promoted the rising incidence rate of physical inactivity for young people (54).

### Limitations

Our study has several limitations commonly described in previously published GBD studies (17, 18). First, despite the application of methods (such as correcting for misclassifications and incompleteness, and redistributing garbage codes) reducing bias in estimates, the data collected from different areas and countries may have several discrepancies in quality, comparability, accuracy, and missing degree, which may result in a certain deviation in the estimation. GBD calculator endeavored to evaluate relative risks that were controlled for confounders. However, as they had to rely on the literature for many relative risks, we did not always have complete control over the choice of confounders controlled for in each study (17). Second, due to the lack of data on gallbladder and biliary disease burden from different ethnicities, we cannot assess the disease burden in across various ethnicities. Our analysis of gallbladder and biliary disease burden mainly concentrated on regional and national levels without the differences between urban and rural areas. Third, the GBD calculator only estimated high BMI attributable to gallbladder and biliary disease burden without considering other metabolic risk factors, such as dyslipidemia and high fasting plasma glucose. The disease burden from other metabolic risk factors may need to be considered in future GBD study frameworks.

## Conclusion

Gallbladder and biliary disease incidence and high BMI attributable to YLDs burden have risen rapidly in global and most SDI regions, especially among people aged 25–49 years. Therefore, high BMI control should be emphasized in the strategic priorities for controlling gallbladder and biliary diseases.

## Data availability statement

The original contributions presented in this study are included in this article/Supplementary material, further inquiries can be directed to the corresponding authors.

## Author contributions

SL, MY, JY, and HL designed the project, edited and revised the manuscript, and supervised the study. JQ, FL, LL, YL, and MZ collected the data and participated in the data analysis. WL and XH performed the statistical analysis. JC, XZ, PZ, and YJ revised the manuscript and gave valuable suggestions for the study design. All authors had approved the final version of this manuscript.

## Footnotes

1
http://ghdx.healthdata.org/gbd-results-tool
