# Supplementary figures and images for: The increasing incidence and high body mass index-related burden of gallbladder and biliary diseases–A results from global burden of disease study 2019
Source: Front Med (Lausanne). 2022 Dec 2;9:1002325. doi: 10.3389/fmed.2022.1002325 (PMC9757069; doi:10.3389/fmed.2022.1002325)

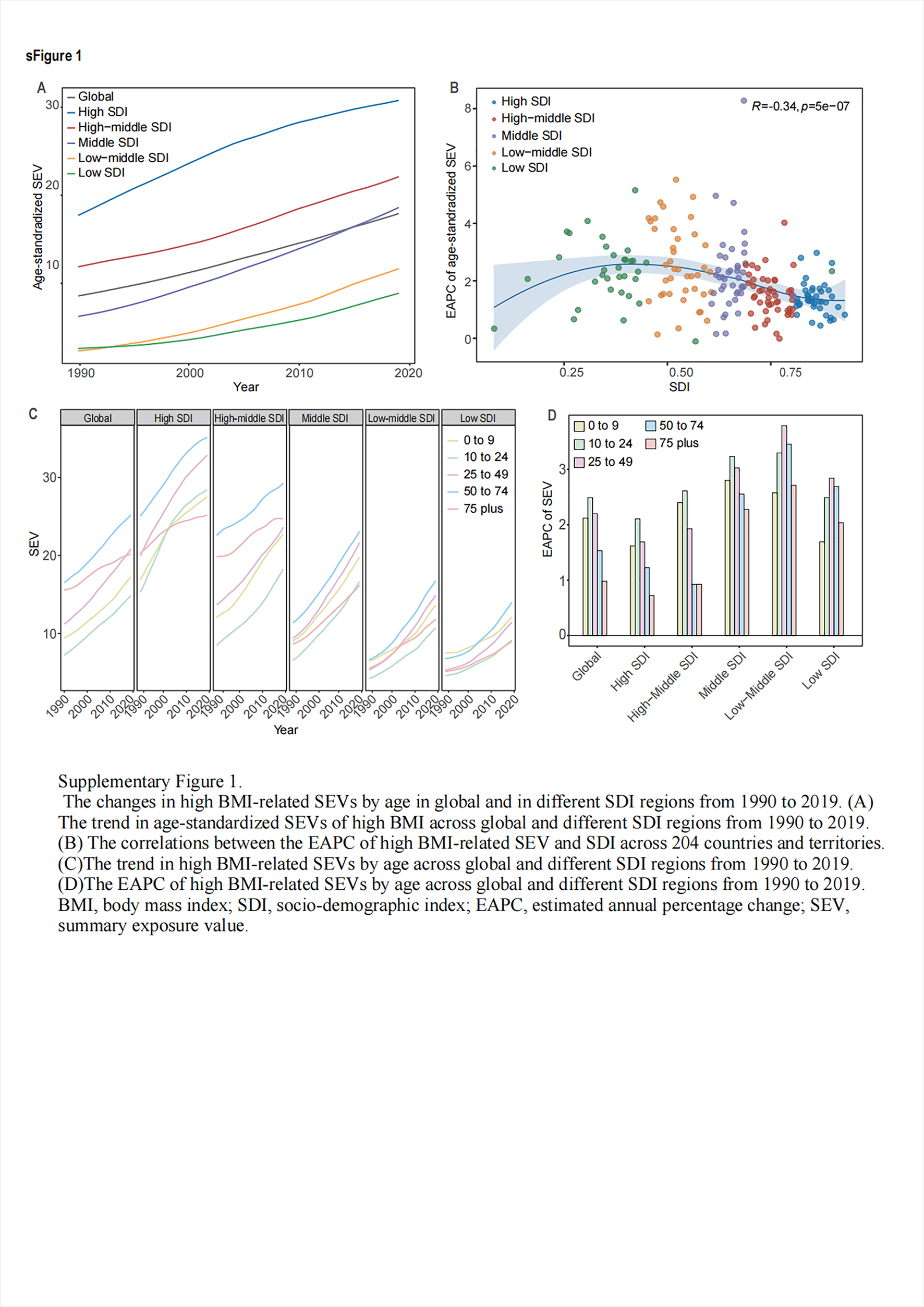

Supplement: Supplementary file 1 [file Image_1.tif]

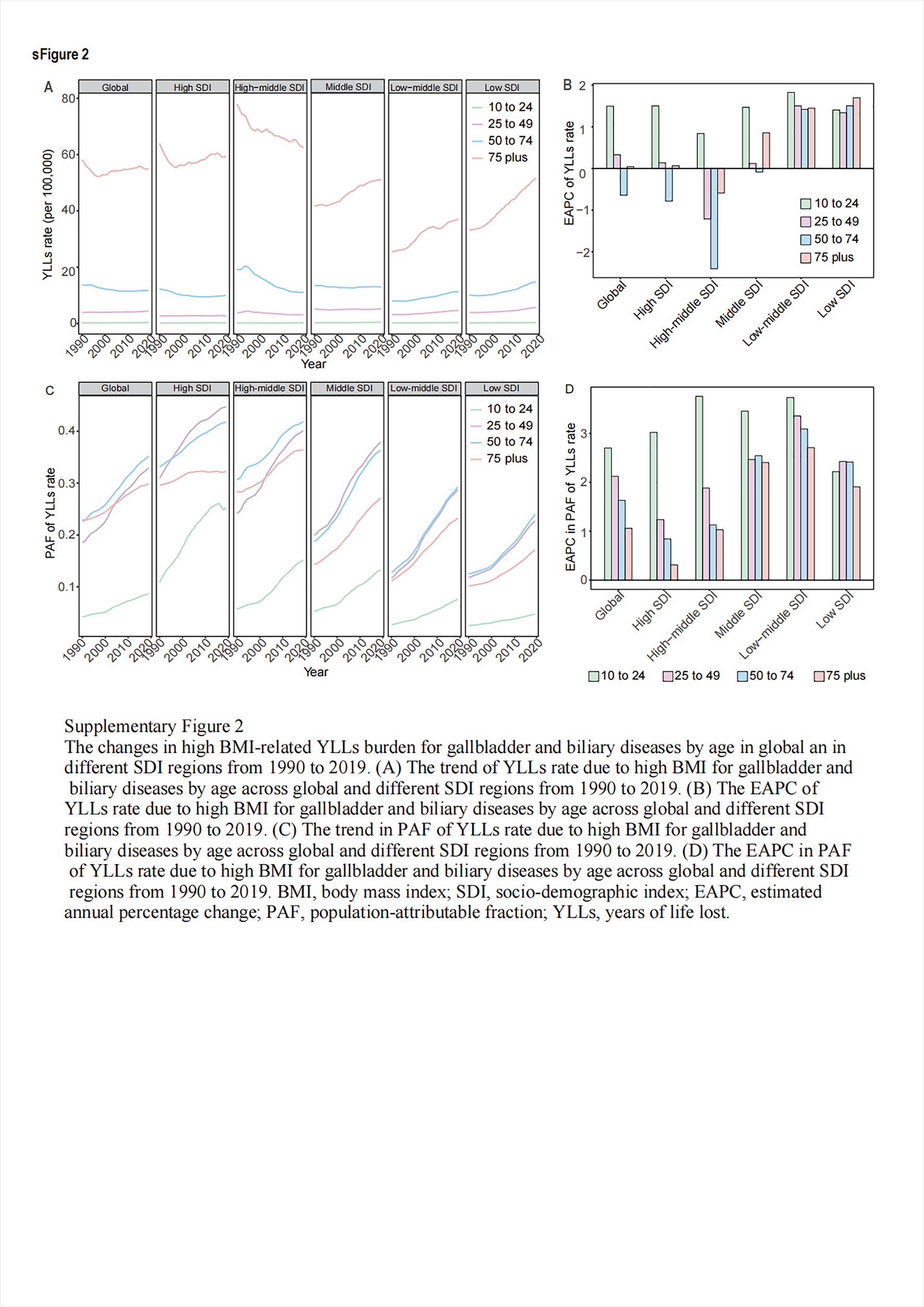

Supplement: Supplementary file 2 [file Image_2.tif]
