# Supplementary material for: The increasing incidence and high body mass index-related burden of gallbladder and biliary diseases–A results from global burden of disease study 2019
Source: Front Med (Lausanne). 2022 Dec 2;9:1002325. doi: 10.3389/fmed.2022.1002325 (PMC9757069; doi:10.3389/fmed.2022.1002325)
Supplement: Supplementary file 3 [file Table_1.pdf]

2Supplementary Tables

Supplementary table 1: The International Obesity Task Force standards of overweight and obesity for children by sex between 2 and 18 years

Age(years)	Body mass index for overweight (kg/㎡)		Body mass index for obesity (kg/㎡)	
	Males	Females	Males	Females
2	18.41	18.02	20.09	19.81
2.5	18.13	17.76	19.80	19.55
3	17.89	17.56	19.57	19.36
3.5	17.69	17.40	19.39	19.23
4	17.55	17.28	19.29	19.15
4.5	17.47	17.19	19.26	19.12
5	17.42	17.15	19.30	19.17
5.5	17.45	17.20	19.47	19.34
6	17.55	17.34	19.78	19.65
6.5	17.71	17.53	20.23	20.08
7	17.92	17.75	20.63	20.51
7.5	18.16	18.03	21.09	21.01
8	18.44	18.35	21.60	21.57
8.5	18.76	18.69	22.17	22.18
9	19.10	19.07	22.77	22.81

9.5	19.46	19.45	23.39	23.46
10	19.84	19.86	24.00	24.11
10.5	20.20	20.9	24.57	24.77
11	20.55	20.74	25.10	25.42
11.5	20.89	21.20	25.58	26.05
12	21.22	21.68	26.02	26.67
12.5	21.56	22.14	26.43	27.24
13	21.91	22.58	26.84	27.76
13.5	22.27	22.98	27.25	28.20
14	22.62	23.34	27.63	28.57
14.5	22.96	23.66	27.98	28.87
15	23.29	23.94	28.30	29.11
15.5	23.60	24.17	28.60	29.29
16	23.90	24.37	28.88	29.43
16.5	24.19	24.54	29.14	2.56
17	24.46	24.70	29.41	29.69
17.5	24.73	24.85	29.70	29.84
18	25	25	30	30
