# Supplementary material for: The increasing incidence and high body mass index-related burden of gallbladder and biliary diseases–A results from global burden of disease study 2019
Source: Front Med (Lausanne). 2022 Dec 2;9:1002325. doi: 10.3389/fmed.2022.1002325 (PMC9757069; doi:10.3389/fmed.2022.1002325)
Supplement: Supplementary file 4 [file Table_2.pdf]

Supplementary Table 2. The change in summary exposure values due to high body-mass index by age in global and in different SDI regions from 1990 to 2019

Location	Age	Summary exposure value (95%UI)		EAPC of summary exposure value (95%CI)
		1990	2019	
Global	Age-standardized	11.09(7.96,15.23)	19.45(15.57,24.39)	1.99(1.97,2.00)
	0-9 years	9.42(6.81,14.14)	17.26(13.25,23.82)	2.12(2.09,2.15)
	10-24 years	7.22(5.14,10.86)	14.86(11.82,19.79)	2.49(2.42,2.56)
	25-49 years	11.21(6.93,17.12)	20.84(15.54,27.88)	2.20(2.18,2.23)
	50-74 years	16.52(10.67,24.01)	25.23(18.00,34.16)	1.53(1.51,1.55)
	75+ years	15.55(9.93,23.05)	20.21(13.79,28.17)	0.98(0.93,1.03)
High SDI	Age-standardized	19.27(14.41,25.31)	30.94(24.88,37.92)	1.63(1.50,1.76)
High-middle SDI		14.05(10.35,18.86)	23.20(18.43,29.42)	1.81(1.77,1.85)
Middle SDI		9.01(6.27,12.76)	20.07(16.21,24.94)	2.86(2.83,2.90)
Low-middle SDI		5.51(3.62,8.33)	13.86(11.00,17.39)	3.33(3.29,3.38)
Low SDI		5.76(3.74,8.56)	11.37(8.71,14.68)	2.47(2.35,2.60)

SDI= sociodemographic index; UI= uncertainty intervals; CI= confidence intervals; EAPC= estimated annual percentage change;
