# Supplementary material for: The increasing incidence and high body mass index-related burden of gallbladder and biliary diseases–A results from global burden of disease study 2019
Source: Front Med (Lausanne). 2022 Dec 2;9:1002325. doi: 10.3389/fmed.2022.1002325 (PMC9757069; doi:10.3389/fmed.2022.1002325)
Supplement: Supplementary file 5 [file Table_3.pdf]

Supplementary Table 3. The age-standardized rates of incidence, YLDs, YLLs for gallbladder and biliary diseases in 2019, and estimated annual percentage change from 1990 to 2019 across 21 GBD regions

Location	Incidence		YLDs		YLLs	
	ASR per 100,000 (95%UI) in 2019	EAPC of ASR (95%CI)	ASR per 100,000 (95%UI) in 2019	EAPC of ASR (95%CI)	ASR per 100,000 (95%UI) in 2019	EAPC of ASR (95%CI)
Australasia	598.97(502.34,709.38)	0.43(0.36,0.51)	39.72(25.01,59.47)	-0.01(-0.06,0.04)	15.39(12.56,18.17)	-0.81(-0.90,-0.71)
High-income Asia Pacific	1426.23(1211.62,1670.16)	0.72(0.58,0.86)	74.46(47.94,109.98)	-0.51(-0.58,-0.43)	19.74(15.90,22.94)	-1.69(-1.93,-1.46)
High-income North America	833.93(713.75,978.73)	0.45(-0.03,0.94)	48.04(31.53,69.57)	0.45(0.04,0.86)	18.70(16.06,21.27)	-0.43(-0.51,-0.36)
Western Europe	825.76(703.33,973.69)	0.27(0.12,0.42)	59.72(37.88,86.73)	-0.73(-0.92,-0.53)	20.26(16.59,22.39)	-0.64(-0.87,-0.42)
Central Europe	1009.87(881.18,1171.35)	-0.12(-0.29,0.04)	75.70(48.70,111.50)	-0.91(-1.00,-0.82)	19.96(17.02,24.48)	-2.40(-2.82,-1.99)
East Asia	958.77(807.46,1136.76)	1.22(1.02,1.42)	71.23(45.29,105.06)	-0.96(-1.10,-0.82)	15.67(13.05,20.65)	-4.73(-4.90,-4.57)
Eastern Europe	786.11(662.11,925.36)	-0.04(-0.09,0.02)	50.84(31.97,75.33)	-0.90(-0.95,-0.86)	22.68(19.41,31.80)	-2.33(-2.56,-2.10)
Southern Latin America	180.13(156.69,214.88)	-1.06(-1.43,-0.68)	15.80(10.01,23.79)	-1.76(-2.14,-1.38)	43.78(37.00,56.34)	-1.91(-2.31,-1.51)
Andean Latin America	250.51(218.69,293.54)	-0.49(-0.67,-0.30)	26.06(16.63,39.01)	-2.00(-2.23,-1.77)	60.72(47.94,77.06)	-2.05(-2.40,-1.71)
Caribbean	313.06(274.27,364.60)	0.03(-0.01,0.08)	34.75(22.19,51.53)	-0.52(-0.61,-0.43)	32.32(24.03,40.92)	-1.63(-2.01,-1.26)
Central Asia	425.29(371.01,499.57)	0.20(0.16,0.24)	44.33(28.15,66.05)	-0.60(-0.64,-0.55)	22.06(19.00,26.95)	-1.78(-1.94,-1.61)
Central Latin America	1112.70(958.42,1295.35)	0.89(0.68,1.11)	100.62(64.01,149.28)	-0.15(-0.26,-0.04)	57.79(47.78,67.28)	-0.45(-0.75,-0.15)

North Africa and Middle East	291.01(251.96,340.51)	0.75(0.55,0.94)	26.06(16.83,38.49)	-0.55(-0.66,-0.43)	32.51(24.92,40.83)	-0.89(-1.00,-0.77)
Southeast Asia	264.47(227.71,309.85)	0.58(0.47,0.68)	26.17(16.57,38.30)	-0.59(-0.72,-0.46)	48.18(35.41,55.65)	-1.62(-1.69,-1.55)
Southern Sub-Saharan Africa	109.99(94.01,129.39)	0.37(0.32,0.41)	11.94(7.59,17.70)	-0.33(-0.46,-0.19)	52.93(42.67,60.40)	0.20(-0.14,0.54)
Tropical Latin America	1041.01(872.81,1229.28)	1.77(1.42,2.13)	80.37(51.08,119.74)	0.27(0.13,0.42)	61.12(49.28,66.60)	0.06(-0.03,0.14)
Central Sub-Saharan Africa	47.61(42.28,55.59)	0.76(0.72,0.79)	8.26(5.31,12.43)	-0.41(-0.46,-0.36)	78.09(52.44,104.05)	-0.36(-0.42,-0.30)
Oceania	142.32(124.13,166.24)	0.26(0.24,0.29)	21.48(13.55,31.88)	-0.15(-0.17,-0.13)	58.34(39.50,91.26)	0.29(0.21,0.38)
South Asia	330.26(280.99,388.41)	2.82(2.38,3.27)	36.54(23.17,53.78)	1.36(0.93,1.78)	16.95(13.03,23.80)	-2.76(-2.95,-2.57)
Eastern Sub-Saharan Africa	51.74(45.46,59.84)	0.94(0.90,0.97)	7.55(4.89,11.12)	-0.57(-0.62,-0.52)	88.41(62.06,143.80)	-0.30(-0.35,-0.25)
Western Sub-Saharan Africa	52.30(45.09,60.88)	0.69(0.67,0.71)	6.80(4.33,9.99)	-0.25(-0.29,-0.21)	60.05(38.04,85.42)	0.46(0.32,0.60)

GBD=Global Burden of Disease; ASR=age-standardized rate； SDI= sociodemographic index; YLDs= years lived with disability; YLLs= years of life lost; UI= uncertainty intervals; CI= confidence intervals; EAPC= estimated annual percentage change;
