# Supplementary material for: The increasing incidence and high body mass index-related burden of gallbladder and biliary diseases–A results from global burden of disease study 2019
Source: Front Med (Lausanne). 2022 Dec 2;9:1002325. doi: 10.3389/fmed.2022.1002325 (PMC9757069; doi:10.3389/fmed.2022.1002325)
Supplement: Supplementary file 6 [file Table_4.pdf]

Supplementary Table 4. The age-standardized rates of incidence, YLDs, YLLs for gallbladder and biliary diseases in 2019, and estimated annual percentage change from 1990 to 2019 across 204 countries and territories

Location	Age-standardized incidence rate per 100,000 (96%UI) in 2019	EAPC of age-standardized incidence rate (95%CI)	Age-standardized YLDs rate per 100,000 (96%UI) in 2019	EAPC of age-standardized YLDs rate (95%CI)	Age-standardized YLLs rate per 100,000 (96%UI) in 2019	EAPC of age-standardized YLLs rate (95%CI)
Andorra	404.88(340.77,484.42)	0.19(0.17,0.21)	26.90(16.99,39.92)	-0.22(-0.25,-0.18)	16.55(10.69,25.65)	-0.36(-0.49,-0.23)
Australia	522.83(435.88,625.10)	0.12(0.09,0.15)	34.80(21.91,52.43)	-0.30(-0.35,-0.26)	15.45(12.55,18.93)	-0.92(-1.03,-0.81)
Austria	790.57(676.69,944.41)	-0.80(-1.04,-0.56)	53.25(33.82,79.83)	-1.26(-1.48,-1.05)	11.76(9.34,14.48)	-2.05(-2.57,-1.52)
Belgium	395.64(330.63,476.84)	0.71(0.11,1.31)	26.77(17.16,39.93)	0.21(-0.36,0.79)	20.76(16.95,26.48)	-1.53(-1.64,-1.41)
Bermuda	338.37(283.34,404.14)	0.16(0.10,0.21)	26.53(16.39,39.92)	-0.71(-0.81,-0.60)	9.26(6.73,13.37)	-4.08(-4.62,-3.55)
Brunei Darussalam	1069.80(936.19,1245.07)	0.06(0.02,0.10)	92.65(59.81,136.85)	-0.54(-0.58,-0.50)	64.74(53.62,78.35)	-0.73(-0.83,-0.63)
Canada	661.70(560.57,791.29)	0.14(0.12,0.16)	44.39(27.78,66.82)	-0.27(-0.31,-0.22)	16.35(13.31,19.86)	-0.93(-1.08,-0.77)
Cyprus	343.02(287.39,415.44)	0.71(0.13,1.30)	23.07(14.56,34.12)	-0.16(-0.70,0.38)	31.25(19.67,38.76)	-2.35(-2.50,-2.20)
Czechia	1196.04(1028.39,1414.16)	0.04(-0.01,0.08)	85.96(54.46,126.99)	-0.67(-0.76,-0.57)	27.34(20.66,35.08)	-2.02(-2.79,-1.25)
Denmark	550.30(460.02,659.95)	-0.92(-1.04,-0.81)	37.49(23.78,56.35)	-1.52(-1.64,-1.41)	19.31(15.46,23.51)	-0.82(-1.18,-0.45)
Estonia	908.51(765.73,1082.44)	-0.15(-0.19,-0.11)	69.63(43.92,103.77)	-1.11(-1.18,-1.04)	12.96(9.09,19.35)	-4.16(-4.55,-3.76)
Finland	455.34(383.64,552.65)	-0.30(-0.99,0.40)	30.14(19.09,45.70)	-0.88(-1.52,-0.23)	16.84(12.57,20.68)	-2.47(-2.61,-2.33)
France	371.36(315.88,448.88)	-0.99(-1.20,-0.79)	24.75(15.41,37.16)	-1.55(-1.78,-1.32)	15.80(12.62,20.46)	-1.84(-1.97,-1.71)
Georgia	468.72(394.73,566.28)	0.87(0.69,1.05)	46.65(29.29,69.93)	0.76(0.55,0.97)	8.28(6.09,11.06)	-0.37(-0.75,0.01)

Germany	627.85(533.56,763.45)	-0.31(-0.88,0.26)	42.56(26.92,62.39)	-0.80(-1.32,-0.28)	18.51(15.35,22.37)	-1.21(-1.94,-0.47)
Guam	159.14(134.71,191.34)	-0.06(-0.12,0.00)	15.28(9.47,23.08)	-0.11(-0.19,-0.03)	15.73(11.96,20.22)	-0.55(-0.79,-0.31)
Iceland	256.79(214.41,309.06)	0.94(0.53,1.36)	16.78(10.64,24.95)	0.57(0.19,0.96)	12.54(9.85,15.78)	-1.79(-1.94,-1.63)
Ireland	421.80(352.91,506.19)	0.19(0.16,0.22)	27.97(17.42,41.57)	-0.52(-0.57,-0.47)	15.45(12.27,18.86)	-1.01(-1.10,-0.92)
Japan	1614.18(1366.14,1898.78)	1.05(0.87,1.23)	76.96(48.94,114.10)	-0.26(-0.32,-0.19)	18.28(14.82,21.81)	-0.96(-1.14,-0.78)
Kuwait	241.06(204.71,289.54)	0.58(0.48,0.69)	18.46(11.41,27.46)	-0.13(-0.23,-0.02)	13.37(10.26,17.00)	-0.65(-0.92,-0.38)
Latvia	1101.82(943.70,1293.90)	0.17(-0.02,0.37)	91.25(58.65,135.72)	-0.56(-0.76,-0.36)	15.97(11.54,21.81)	-2.83(-3.09,-2.58)
Lithuania	1214.84(1044.90,1417.39)	0.24(0.01,0.47)	100.79(64.28,148.84)	-0.27(-0.50,-0.04)	25.57(17.16,34.05)	-0.55(-0.96,-0.13)
Luxembourg	461.78(385.11,558.27)	-0.05(-0.72,0.63)	30.71(19.20,45.47)	-0.66(-1.27,-0.04)	17.17(13.17,22.77)	-1.66(-1.79,-1.53)
Monaco	426.14(358.89,518.82)	0.24(0.21,0.26)	28.36(17.73,42.90)	-0.16(-0.17,-0.15)	15.72(11.71,19.66)	0.11(-0.07,0.29)
Netherlands	430.35(363.68,526.44)	0.03(0.01,0.05)	28.07(17.74,41.65)	-0.47(-0.50,-0.44)	19.15(15.44,22.92)	-1.17(-1.28,-1.07)
New Zealand	1020.31(852.88,1219.84)	1.44(1.19,1.68)	67.45(42.34,100.06)	0.98(0.71,1.25)	15.02(9.17,18.55)	-0.21(-0.37,-0.05)
Norway	1186.13(989.83,1434.13)	1.30(0.88,1.72)	85.69(52.79,128.39)	-0.20(-0.26,-0.13)	16.33(12.66,18.89)	-2.01(-2.25,-1.77)
Puerto Rico	356.97(302.13,421.43)	0.00(-0.04,0.04)	28.69(18.03,42.47)	-0.72(-0.79,-0.66)	23.02(16.60,31.17)	-2.49(-2.74,-2.24)
Qatar	188.25(160.39,225.51)	0.25(0.17,0.32)	14.39(9.10,21.33)	-1.22(-1.31,-1.13)	17.84(12.86,25.53)	0.15(-0.17,0.48)
Republic of Korea	1040.40(878.58,1230.76)	-0.17(-0.23,-0.11)	70.86(44.70,105.60)	-1.40(-1.53,-1.28)	28.95(21.44,35.08)	-3.83(-4.06,-3.59)
Republic of Moldova	826.20(707.69,975.37)	-0.22(-0.28,-0.16)	78.15(49.91,118.04)	-0.97(-1.03,-0.91)	14.97(11.71,19.65)	-3.18(-3.64,-2.71)
San Marino	382.12(315.77,459.03)	0.23(0.21,0.26)	25.78(16.11,39.20)	-0.18(-0.24,-0.13)	7.05(4.49,10.55)	-0.32(-0.48,-0.16)

Singapore	999.26(850.11,1193.72)	0.04(0.01,0.06)	69.35(43.78,103.26)	-0.73(-0.75,-0.70)	16.17(13.01,19.35)	-2.58(-2.72,-2.43)
Slovakia	1411.50(1219.56,1628.64)	-0.28(-0.36,-0.20)	109.65(69.65,162.57)	-1.02(-1.11,-0.93)	28.09(21.31,37.35)	-1.70(-2.05,-1.36)
Slovenia	1074.36(925.57,1266.17)	0.34(0.21,0.48)	74.46(47.44,111.60)	-0.46(-0.58,-0.35)	21.39(13.22,29.22)	-1.66(-1.92,-1.40)
Sweden	717.09(593.14,870.44)	0.62(0.30,0.95)	37.03(23.16,56.22)	0.20(-0.10,0.50)	15.47(12.78,19.32)	-1.02(-1.25,-0.80)
Switzerland	416.49(349.26,504.51)	0.09(-0.71,0.89)	27.08(17.13,40.99)	-0.32(-1.09,0.46)	11.67(7.58,14.88)	0.02(-0.17,0.22)
Taiwan (Province of China)	238.18(203.99,281.14)	-0.21(-0.38,-0.04)	17.04(10.65,25.05)	-0.97(-1.19,-0.74)	33.16(24.94,43.17)	-2.33(-2.48,-2.18)
United Arab Emirates	176.77(152.50,208.88)	0.14(0.08,0.20)	17.39(11.10,25.99)	-0.72(-0.79,-0.66)	20.47(9.18,32.62)	-0.98(-1.59,-0.37)
United Kingdom	1593.45(1339.08,1878.10)	1.09(0.76,1.42)	136.51(86.40,199.28)	-0.13(-0.79,0.53)	27.86(19.69,30.60)	1.60(1.46,1.75)
American Samoa	188.61(163.13,220.01)	0.49(0.43,0.55)	20.21(13.02,30.14)	0.09(0.04,0.14)	62.36(46.22,76.78)	0.82(0.40,1.23)
Antigua and Barbuda	332.53(285.81,393.53)	0.08(0.03,0.13)	31.77(20.28,47.01)	-0.56(-0.63,-0.49)	25.49(19.72,33.09)	-1.57(-1.98,-1.16)
Argentina	178.23(153.92,212.94)	-1.39(-1.91,-0.86)	16.23(10.11,24.44)	-1.98(-2.49,-1.47)	46.09(37.57,59.87)	-1.49(-1.94,-1.04)
Armenia	520.31(447.77,612.70)	0.61(0.55,0.67)	47.28(29.94,71.41)	-0.36(-0.40,-0.31)	41.00(26.40,52.40)	0.33(-0.16,0.83)
Bahamas	341.76(293.28,402.89)	-0.09(-0.12,-0.05)	35.04(22.58,51.94)	-0.58(-0.67,-0.49)	40.27(29.69,53.90)	-1.39(-1.71,-1.07)
Bahrain	235.20(196.82,279.79)	-0.34(-0.45,-0.23)	19.59(12.46,29.27)	-1.62(-1.72,-1.52)	30.29(22.96,41.44)	-3.81(-4.29,-3.33)
Barbados	315.49(266.22,372.33)	0.06(0.01,0.11)	29.84(18.68,45.31)	-0.54(-0.62,-0.45)	17.46(12.30,23.81)	-1.79(-2.09,-1.48)
Belarus	942.32(813.50,1116.13)	0.10(0.04,0.16)	76.71(49.06,114.95)	-0.74(-0.76,-0.72)	20.05(13.76,30.03)	-2.07(-2.35,-1.79)
Bosnia and Herzegovina	1022.15(882.82,1193.92)	0.37(0.34,0.41)	87.15(56.61,127.41)	-0.81(-0.87,-0.75)	21.03(15.79,28.46)	-1.53(-1.73,-1.33)
Bulgaria	967.44(816.58,1142.57)	0.11(0.02,0.19)	83.17(52.99,125.28)	-0.45(-0.52,-0.38)	14.55(10.32,20.74)	-1.09(-1.70,-0.47)

Chile	184.24(160.68,216.48)	-0.43(-0.51,-0.36)	14.82(9.51,22.00)	-1.37(-1.50,-1.24)	38.82(31.64,52.00)	-3.45(-3.78,-3.11)
China	983.15(827.97,1166.37)	1.23(1.03,1.44)	72.87(46.34,107.47)	-0.97(-1.11,-0.83)	15.06(12.37,20.21)	-4.89(-5.06,-4.72)
Cook Islands	185.82(159.63,220.99)	0.28(0.25,0.31)	16.77(10.58,24.86)	-0.18(-0.22,-0.13)	66.69(49.96,85.66)	-1.64(-1.78,-1.51)
Croatia	1104.42(951.98,1287.42)	0.47(0.35,0.59)	79.90(50.91,119.16)	-0.20(-0.30,-0.10)	19.40(13.38,26.01)	-0.60(-0.90,-0.29)
Dominica	278.65(239.52,326.32)	-0.23(-0.28,-0.18)	31.13(19.89,46.42)	-0.55(-0.65,-0.44)	18.79(14.06,24.68)	-1.94(-2.25,-1.63)
Greece	420.18(358.88,506.27)	0.38(0.33,0.44)	29.06(18.42,43.12)	0.03(-0.07,0.13)	18.72(12.62,23.15)	1.50(0.92,2.08)
Greenland	625.24(544.37,726.40)	0.06(0.02,0.09)	59.62(38.80,88.50)	-0.49(-0.53,-0.46)	35.46(24.35,45.33)	-1.91(-2.14,-1.68)
Hungary	1173.54(1012.90,1383.65)	-0.09(-0.13,-0.05)	91.44(57.95,134.88)	-0.81(-0.90,-0.73)	32.15(24.84,41.29)	-1.98(-2.43,-1.53)
Israel	430.85(360.90,516.62)	0.27(0.22,0.32)	30.48(19.45,45.43)	-0.37(-0.39,-0.34)	26.61(18.03,32.49)	0.26(-0.10,0.63)
Italy	1718.36(1441.49,2039.23)	0.69(0.51,0.87)	115.01(72.88,168.92)	-0.90(-1.33,-0.47)	18.34(15.84,23.27)	-1.06(-1.31,-0.80)
Jordan	253.78(215.19,304.72)	0.12(0.08,0.17)	22.17(14.07,32.84)	-1.07(-1.12,-1.02)	28.38(19.83,36.59)	-2.28(-2.41,-2.15)
Kazakhstan	447.71(388.36,533.56)	-0.01(-0.08,0.06)	43.48(27.73,64.91)	-1.03(-1.21,-0.84)	22.69(17.94,29.67)	-3.14(-3.78,-2.48)
Lebanon	273.77(230.73,328.53)	0.84(0.80,0.88)	21.22(13.17,31.87)	-0.49(-0.57,-0.42)	17.27(8.47,28.69)	-0.05(-0.24,0.13)
Libya	231.38(196.91,276.53)	0.44(0.42,0.47)	21.67(13.56,32.24)	-0.43(-0.50,-0.37)	20.09(12.54,29.12)	-0.43(-0.62,-0.24)
Malaysia	266.68(226.32,320.37)	0.42(0.39,0.46)	25.20(15.81,38.55)	-0.69(-0.74,-0.64)	62.63(46.15,81.41)	-0.17(-0.42,0.07)
Malta	202.70(170.50,241.94)	0.89(0.48,1.30)	14.15(8.85,21.10)	0.36(-0.02,0.74)	15.02(11.69,18.79)	-1.40(-1.50,-1.30)
Mauritius	224.15(188.05,269.61)	0.40(0.34,0.47)	21.21(13.38,32.15)	-0.18(-0.27,-0.09)	15.93(10.36,21.04)	0.66(0.23,1.08)
Montenegro	955.05(812.06,1132.45)	0.28(0.23,0.32)	76.63(48.82,113.06)	-0.27(-0.30,-0.24)	6.53(5.16,8.21)	-0.15(-0.33,0.02)

Niue	191.39(166.79,226.19)	0.16(0.15,0.18)	17.60(11.35,26.33)	-0.24(-0.26,-0.22)	61.78(42.50,83.78)	-0.61(-0.67,-0.54)
North Macedonia	938.02(788.00,1105.05)	0.27(0.23,0.31)	78.68(50.34,115.51)	-0.67(-0.72,-0.61)	10.01(7.75,13.30)	-1.76(-1.94,-1.58)
Northern Mariana Islands	232.95(203.24,275.31)	0.29(0.21,0.38)	20.10(12.94,29.58)	0.02(-0.01,0.05)	72.90(51.55,91.83)	-0.73(-0.94,-0.52)
Oman	209.63(175.43,250.43)	0.24(0.10,0.37)	17.34(10.77,25.63)	-0.81(-0.91,-0.71)	10.99(5.88,14.90)	-0.88(-1.03,-0.73)
Palau	190.75(165.66,226.40)	0.13(0.10,0.16)	17.76(11.38,26.48)	-0.32(-0.37,-0.26)	57.59(34.84,80.91)	-0.18(-0.26,-0.10)
Panama	840.98(718.22,994.62)	0.05(0.00,0.09)	79.89(50.08,119.35)	-0.56(-0.64,-0.49)	22.75(15.75,32.58)	-2.42(-2.74,-2.11)
Poland	804.82(683.53,948.49)	-0.88(-1.40,-0.36)	49.63(32.43,72.31)	-1.67(-2.10,-1.23)	18.16(14.55,25.87)	-3.51(-3.92,-3.10)
Portugal	218.27(186.89,259.21)	1.60(1.37,1.82)	15.35(9.67,22.68)	0.74(0.48,1.00)	23.07(15.44,28.11)	0.11(-0.06,0.28)
Romania	1142.91(985.76,1336.64)	0.33(0.23,0.42)	96.44(61.01,144.40)	-0.61(-0.69,-0.54)	13.58(9.71,21.44)	-3.14(-3.66,-2.61)
Russian Federation	693.51(581.75,813.24)	0.09(0.01,0.16)	42.67(26.78,63.66)	-0.91(-0.96,-0.86)	25.89(21.63,36.29)	-2.30(-2.55,-2.05)
Saint Kitts and Nevis	359.67(313.45,427.61)	-0.47(-0.56,-0.38)	32.90(20.88,49.17)	-1.16(-1.31,-1.01)	43.08(29.86,64.04)	-3.21(-3.74,-2.68)
Saudi Arabia	250.78(215.73,299.84)	0.23(0.17,0.29)	20.83(13.35,31.21)	-1.05(-1.08,-1.02)	34.44(26.31,45.62)	-2.60(-2.75,-2.46)
Serbia	960.08(822.46,1135.71)	0.59(0.50,0.67)	78.37(49.77,114.00)	-0.34(-0.40,-0.28)	23.43(17.78,29.60)	-0.10(-0.25,0.06)
Seychelles	209.60(177.68,249.43)	0.32(0.28,0.37)	21.59(13.49,32.70)	-0.57(-0.65,-0.49)	17.40(10.37,24.47)	-0.41(-0.57,-0.24)
Spain	303.23(254.59,364.82)	-0.47(-0.66,-0.27)	19.93(12.47,30.54)	-1.11(-1.34,-0.88)	27.34(18.84,33.33)	-0.38(-0.51,-0.26)
Sri Lanka	224.79(187.70,276.03)	0.70(0.61,0.78)	20.70(12.99,31.41)	-0.49(-0.58,-0.40)	6.28(4.45,8.76)	-1.77(-1.96,-1.59)
Thailand	251.09(216.91,294.46)	-0.60(-0.80,-0.39)	22.28(13.93,32.29)	-1.90(-2.17,-1.62)	51.21(35.64,68.72)	-2.11(-2.28,-1.95)
Trinidad and Tobago	308.15(265.41,367.97)	0.03(-0.02,0.07)	32.28(20.09,48.46)	-0.72(-0.80,-0.64)	26.31(17.61,36.85)	-1.94(-2.23,-1.66)

Turkey	295.65(254.13,350.54)	1.66(1.24,2.08)	24.69(15.86,36.93)	-0.06(-0.39,0.28)	28.60(20.42,36.38)	-1.51(-1.70,-1.32)
Ukraine	1000.51(812.99,1216.21)	-0.16(-0.21,-0.10)	63.56(39.29,95.18)	-0.82(-0.88,-0.75)	14.56(10.75,23.51)	-2.88(-3.32,-2.44)
United States Virgin Islands	366.75(315.46,433.43)	0.13(0.09,0.17)	34.27(21.60,51.26)	-0.53(-0.60,-0.46)	43.30(32.15,55.15)	-1.07(-1.29,-0.84)
Uruguay	181.11(157.13,215.07)	0.24(0.20,0.28)	15.85(10.18,23.68)	-0.35(-0.41,-0.29)	42.87(33.00,51.38)	-0.23(-0.57,0.11)
Albania	959.81(802.71,1152.85)	0.63(0.60,0.66)	79.28(49.72,118.59)	-0.46(-0.51,-0.41)	8.29(5.88,11.63)	-1.81(-2.11,-1.50)
Algeria	239.49(202.88,288.59)	0.31(0.30,0.32)	22.38(14.43,33.86)	-0.72(-0.74,-0.69)	19.91(13.35,26.55)	-1.15(-1.25,-1.05)
Azerbaijan	399.76(344.60,472.53)	0.16(0.10,0.23)	42.06(26.97,62.98)	-0.85(-0.88,-0.81)	16.85(10.81,34.01)	-2.69(-2.99,-2.39)
Botswana	114.87(100.30,136.60)	1.35(0.40,2.30)	13.91(8.84,20.87)	0.27(-0.70,1.25)	88.02(57.74,133.23)	-0.61(-1.09,-0.14)
Brazil	1052.16(880.38,1243.88)	1.80(1.45,2.16)	80.68(51.25,120.37)	0.29(0.14,0.44)	61.44(49.45,67.02)	0.06(-0.03,0.14)
Colombia	979.36(833.57,1154.72)	0.60(0.56,0.64)	89.54(55.90,134.09)	-0.52(-0.57,-0.48)	46.91(32.83,63.94)	-0.34(-0.58,-0.10)
Costa Rica	948.29(817.05,1117.06)	0.27(0.22,0.32)	83.50(53.86,124.99)	-0.36(-0.44,-0.28)	36.78(26.36,48.59)	-0.93(-1.36,-0.50)
Cuba	344.97(295.76,408.87)	-0.10(-0.17,-0.04)	29.69(18.48,43.95)	-0.68(-0.78,-0.59)	24.46(18.35,32.85)	-2.30(-2.78,-1.83)
Ecuador	265.67(227.54,312.22)	0.78(0.73,0.82)	27.23(17.17,40.55)	-0.66(-0.80,-0.51)	73.08(50.78,95.24)	0.08(-0.62,0.77)
Egypt	299.89(261.76,351.83)	0.29(0.25,0.33)	30.32(19.40,46.10)	-0.90(-0.96,-0.84)	73.87(42.70,112.91)	-0.66(-0.81,-0.50)
Equatorial Guinea	52.55(45.72,61.10)	1.41(1.23,1.58)	7.00(4.40,10.48)	-1.69(-1.77,-1.61)	67.75(40.26,105.57)	-1.73(-1.85,-1.61)
Fiji	156.12(136.68,182.30)	0.36(0.29,0.42)	19.96(12.54,29.28)	-0.04(-0.11,0.02)	44.24(29.46,59.64)	1.40(1.04,1.77)
Gabon	48.94(42.53,57.47)	0.49(0.48,0.51)	6.72(4.10,9.97)	-0.59(-0.62,-0.56)	66.37(39.76,122.60)	-0.54(-0.78,-0.30)
Grenada	310.52(267.97,368.80)	-0.07(-0.13,-0.01)	33.28(21.13,50.09)	-0.76(-0.90,-0.63)	33.29(25.64,44.55)	-1.96(-2.32,-1.59)

Guyana	314.48(271.99,365.91)	0.02(-0.05,0.08)	40.42(25.64,61.10)	-0.63(-0.72,-0.54)	50.42(35.75,68.38)	-1.36(-1.79,-0.94)
Indonesia	253.32(212.97,299.01)	0.76(0.65,0.87)	24.81(15.61,36.74)	-0.32(-0.45,-0.19)	64.40(41.28,77.44)	-1.32(-1.43,-1.20)
Iran (Islamic Republic of)	484.21(405.67,575.14)	0.74(0.32,1.17)	31.92(20.27,47.71)	-0.25(-0.60,0.10)	19.69(12.43,23.76)	0.26(-0.07,0.59)
Iraq	220.83(187.29,263.89)	0.42(0.36,0.47)	22.16(13.95,33.35)	-0.78(-0.80,-0.75)	12.11(8.93,15.61)	-2.20(-2.32,-2.07)
Jamaica	296.02(254.77,345.33)	0.09(0.02,0.16)	30.12(19.02,44.49)	-0.34(-0.46,-0.22)	16.41(11.54,22.75)	-0.90(-1.49,-0.31)
Mexico	1288.75(1082.77,1519.90)	1.21(0.86,1.56)	108.62(68.98,163.54)	0.24(0.05,0.43)	67.18(52.34,80.33)	-0.08(-0.42,0.25)
Namibia	108.87(95.14,127.61)	0.70(0.65,0.75)	14.66(9.26,21.42)	-0.77(-0.84,-0.71)	82.70(53.81,130.44)	-0.54(-0.78,-0.30)
Nauru	192.83(168.01,223.02)	0.33(0.29,0.37)	21.97(14.11,32.54)	0.04(-0.07,0.14)	96.41(63.06,135.91)	-0.23(-0.47,0.01)
Paraguay	658.74(570.45,778.38)	0.47(0.43,0.51)	70.23(44.88,104.69)	-0.42(-0.48,-0.36)	48.66(32.98,64.97)	0.04(-0.31,0.39)
Peru	247.67(213.05,293.78)	-1.23(-1.52,-0.93)	24.32(15.36,35.99)	-2.79(-3.13,-2.45)	46.38(32.21,66.73)	-3.29(-3.64,-2.94)
Philippines	360.07(303.77,427.99)	0.94(0.79,1.10)	33.64(21.15,49.49)	0.38(0.20,0.56)	37.51(29.96,50.55)	-1.01(-1.22,-0.81)
Saint Lucia	295.36(248.70,353.03)	0.06(0.00,0.12)	30.41(19.29,45.29)	-0.73(-0.86,-0.60)	20.80(15.38,27.69)	-2.37(-2.92,-1.81)
Saint Vincent and the Grenadines	293.88(254.62,345.36)	-0.14(-0.19,-0.09)	32.94(21.02,49.35)	-0.64(-0.74,-0.54)	30.49(23.51,38.67)	-1.43(-1.91,-0.94)
Samoa	181.70(157.99,213.29)	0.19(0.15,0.23)	21.33(13.56,31.89)	-0.25(-0.28,-0.21)	65.68(48.14,88.32)	-0.36(-0.46,-0.27)
South Africa	115.51(97.64,137.25)	0.34(0.29,0.38)	10.90(6.96,16.13)	-0.50(-0.64,-0.37)	45.47(32.41,53.40)	-0.09(-0.50,0.33)
Suriname	310.12(266.68,367.00)	0.04(-0.02,0.09)	37.38(23.88,55.72)	-0.69(-0.75,-0.63)	43.43(33.14,55.85)	-1.83(-2.20,-1.46)
Syrian Arab Republic	239.68(202.46,286.38)	0.50(0.47,0.53)	22.06(13.87,32.73)	-0.74(-0.85,-0.62)	13.87(7.99,19.61)	-1.90(-2.15,-1.64)

Tokelau	167.92(145.90,198.67)	0.31(0.27,0.36)	19.62(12.46,29.38)	-0.63(-0.68,-0.58)	59.83(42.73,81.11)	-0.82(-0.87,-0.78)
Tonga	191.25(166.95,226.24)	0.09(0.06,0.12)	22.38(14.13,32.77)	-0.39(-0.43,-0.35)	76.48(55.53,101.28)	-0.43(-0.54,-0.32)
Tunisia	161.63(137.52,192.15)	-0.13(-0.44,0.19)	13.76(8.76,20.26)	-1.16(-1.48,-0.84)	19.04(11.52,29.78)	0.34(0.21,0.48)
Turkmenistan	393.66(340.09,458.86)	0.19(0.15,0.23)	42.74(27.50,63.18)	-0.70(-0.73,-0.67)	20.33(14.12,28.90)	-1.53(-1.76,-1.30)
Uzbekistan	423.21(362.16,500.79)	0.40(0.36,0.43)	44.27(28.07,66.34)	-0.36(-0.39,-0.33)	23.47(18.40,29.99)	0.12(-0.17,0.42)
Viet Nam	262.78(223.73,310.73)	0.58(0.50,0.66)	25.93(16.26,39.45)	-0.80(-0.91,-0.70)	35.80(25.19,47.44)	-2.71(-2.93,-2.48)
Angola	46.58(41.01,53.77)	1.25(1.21,1.30)	7.93(5.00,12.10)	-0.65(-0.69,-0.60)	81.28(49.93,126.62)	-0.06(-0.15,0.03)
Bangladesh	136.60(115.88,159.85)	1.21(1.16,1.26)	17.13(10.92,25.75)	-0.53(-0.58,-0.49)	19.01(12.42,31.76)	-3.03(-3.20,-2.86)
Belize	320.55(276.44,371.21)	0.24(0.20,0.28)	36.32(22.89,53.82)	-0.36(-0.41,-0.31)	40.14(31.23,50.76)	-0.79(-0.93,-0.65)
Bhutan	129.86(111.14,153.83)	0.92(0.90,0.94)	15.78(9.90,23.26)	-1.31(-1.40,-1.22)	18.34(9.47,35.80)	-2.03(-2.15,-1.92)
Bolivia (Plurinational State of)	236.44(205.73,273.80)	0.62(0.58,0.66)	29.95(19.30,45.20)	-1.23(-1.28,-1.17)	94.63(63.46,137.34)	-1.55(-1.68,-1.43)
Cabo Verde	45.50(37.54,53.90)	0.49(0.47,0.51)	5.23(3.28,7.82)	-0.82(-0.87,-0.77)	25.13(19.93,30.58)	-0.77(-1.04,-0.49)
Cambodia	225.71(195.28,269.23)	0.66(0.63,0.69)	31.20(19.75,46.73)	-1.17(-1.23,-1.11)	56.18(37.51,83.15)	-1.48(-1.59,-1.36)
Cameroon	43.58(37.54,50.84)	0.60(0.57,0.63)	7.04(4.46,10.55)	-0.58(-0.62,-0.53)	49.29(30.67,71.79)	-0.37(-0.62,-0.11)
Comoros	51.86(45.85,60.22)	0.93(0.87,1.00)	8.25(5.22,12.41)	-0.70(-0.74,-0.66)	96.32(57.32,159.18)	-0.41(-0.56,-0.26)
Congo	46.49(40.50,53.68)	0.81(0.75,0.88)	7.45(4.69,11.33)	-0.72(-0.75,-0.68)	81.25(49.94,121.72)	-0.34(-0.54,-0.14)
Democratic People's Republic of Korea	307.14(264.47,359.36)	-0.31(-0.36,-0.26)	33.23(21.56,50.06)	-0.63(-0.68,-0.58)	27.62(16.40,39.94)	-1.70(-1.77,-1.63)

Democratic Republic of the Congo	48.26(42.45,56.49)	0.67(0.63,0.70)	8.32(5.35,12.52)	-0.32(-0.37,-0.26)	74.00(47.86,103.49)	-0.48(-0.54,-0.41)
Djibouti	45.01(38.97,52.92)	0.63(0.57,0.69)	6.80(4.26,10.30)	-0.79(-0.84,-0.74)	65.90(37.49,116.27)	-0.30(-0.38,-0.21)
Dominican Republic	276.88(237.37,326.21)	0.33(0.29,0.38)	31.45(20.17,47.06)	-0.53(-0.65,-0.40)	21.92(14.12,31.16)	-0.72(-1.38,-0.06)
El Salvador	929.11(796.62,1097.83)	0.30(0.25,0.35)	95.50(60.58,142.30)	-0.93(-1.09,-0.77)	40.66(30.03,54.98)	-2.45(-3.22,-1.67)
Eswatini	96.66(84.84,112.61)	0.34(0.29,0.39)	15.26(9.75,22.86)	-0.31(-0.42,-0.19)	85.94(56.37,130.05)	0.92(0.38,1.47)
Ghana	46.03(39.78,53.99)	0.74(0.70,0.77)	6.84(4.19,10.22)	-0.16(-0.21,-0.11)	66.24(43.74,85.74)	1.25(0.93,1.57)
Guatemala	882.89(765.22,1047.50)	0.63(0.59,0.67)	107.74(68.32,160.96)	-1.02(-1.20,-0.84)	52.67(39.40,70.96)	-1.99(-2.83,-1.14)
Honduras	1038.04(906.14,1213.89)	0.54(0.49,0.59)	128.37(82.34,192.13)	-0.62(-0.66,-0.59)	164.76(116.57,227.61)	0.21(0.05,0.37)
India	377.52(320.64,444.64)	3.07(2.58,3.56)	41.58(26.34,61.52)	1.59(1.11,2.08)	15.97(11.90,23.16)	-2.88(-3.11,-2.64)
Kenya	63.20(53.81,74.96)	0.42(0.37,0.47)	7.15(4.57,10.71)	-0.36(-0.43,-0.29)	100.58(58.41,160.75)	0.53(0.42,0.65)
Kiribati	166.80(146.55,195.44)	0.33(0.31,0.35)	30.35(19.43,44.46)	-0.28(-0.31,-0.25)	131.77(78.53,191.51)	-0.83(-0.88,-0.77)
Kyrgyzstan	400.46(344.52,471.53)	-0.07(-0.13,-0.02)	42.62(26.64,62.57)	-0.94(-0.98,-0.89)	18.72(14.70,26.90)	-2.75(-3.07,-2.44)
Lao People's Democratic Republic	211.14(185.74,246.88)	1.07(1.04,1.09)	32.92(21.01,49.33)	-0.90(-0.95,-0.85)	51.92(26.93,92.34)	-1.43(-1.52,-1.34)
Lesotho	94.21(81.97,109.17)	0.35(0.32,0.39)	17.64(11.20,26.64)	0.02(-0.11,0.14)	109.59(75.10,161.86)	1.75(1.40,2.11)
Maldives	207.10(168.83,252.18)	0.87(0.78,0.95)	18.59(11.38,28.08)	-0.98(-1.04,-0.92)	6.92(5.08,8.62)	-3.55(-3.81,-3.30)
Marshall Islands	169.20(147.53,194.46)	0.22(0.19,0.25)	24.17(15.37,36.06)	-0.43(-0.46,-0.40)	90.22(51.59,135.08)	-0.63(-0.72,-0.54)
Mauritania	42.47(36.29,49.57)	0.62(0.59,0.66)	6.27(3.90,9.46)	-0.76(-0.81,-0.70)	34.44(22.26,47.81)	-1.64(-1.76,-1.53)

Micronesia (Federated States of)	184.34(162.01,214.53)	0.33(0.30,0.37)	24.06(15.25,35.78)	-0.67(-0.70,-0.63)	92.30(55.43,139.44)	-0.88(-1.04,-0.73)
Mongolia	449.51(392.19,525.38)	0.01(-0.03,0.05)	53.36(33.64,80.50)	-1.61(-1.71,-1.51)	46.09(34.19,62.74)	-4.01(-4.37,-3.65)
Morocco	229.91(197.89,278.19)	0.68(0.67,0.70)	25.13(16.01,37.88)	-0.55(-0.58,-0.52)	26.61(19.03,34.41)	-0.03(-0.14,0.08)
Myanmar	200.26(171.92,237.19)	0.62(0.59,0.65)	27.36(17.33,41.37)	-0.96(-1.03,-0.90)	23.71(15.75,32.44)	-1.85(-2.08,-1.61)
Nicaragua	867.53(747.44,1012.95)	0.49(0.43,0.54)	87.64(55.73,128.90)	-0.83(-0.91,-0.75)	41.40(32.09,58.37)	-0.99(-1.20,-0.78)
Nigeria	62.98(53.53,74.02)	0.74(0.71,0.77)	6.19(3.89,9.17)	-0.19(-0.25,-0.14)	62.56(30.30,121.98)	0.57(0.35,0.79)
Palestine	241.47(204.65,282.35)	0.15(0.05,0.24)	23.51(14.92,34.69)	-0.58(-0.65,-0.51)	25.45(13.99,35.19)	-0.83(-0.95,-0.71)
Sao Tome and Principe	41.46(35.21,48.85)	0.22(0.19,0.25)	5.97(3.72,9.08)	-0.85(-0.91,-0.80)	26.43(18.17,50.99)	-1.74(-2.18,-1.30)
Sudan	209.45(179.78,248.17)	0.57(0.53,0.62)	25.75(16.45,38.76)	-0.55(-0.60,-0.50)	29.71(14.94,50.71)	0.42(0.30,0.55)
Tajikistan	370.88(318.93,433.04)	0.29(0.24,0.34)	46.79(29.18,68.97)	-0.36(-0.40,-0.32)	19.56(14.24,27.56)	-1.16(-1.28,-1.04)
Timor-Leste	226.30(196.42,266.90)	1.16(1.12,1.19)	31.39(19.92,47.30)	-0.68(-0.72,-0.63)	51.41(28.29,105.30)	-0.36(-0.52,-0.21)
Tuvalu	175.24(151.28,207.50)	0.27(0.22,0.32)	21.11(13.52,31.82)	-0.63(-0.66,-0.59)	78.07(51.09,112.35)	-0.83(-0.88,-0.77)
Vanuatu	183.39(160.18,216.15)	0.13(0.09,0.18)	28.79(18.32,42.51)	-0.19(-0.24,-0.14)	96.09(51.14,139.37)	-0.38(-0.51,-0.24)
Venezuela (Bolivarian Republic of)	882.71(750.04,1039.04)	0.22(0.20,0.25)	85.44(53.18,127.54)	-0.74(-0.84,-0.63)	32.72(22.90,44.49)	-1.65(-2.06,-1.23)
Zambia	48.08(42.20,56.19)	0.60(0.56,0.64)	7.33(4.63,11.03)	-0.95(-1.02,-0.87)	83.71(51.65,117.70)	-0.82(-1.07,-0.57)
Zimbabwe	86.69(75.04,103.08)	0.00(-0.19,0.18)	15.62(9.78,23.34)	0.42(0.35,0.48)	73.33(39.47,116.57)	1.60(1.36,1.84)
Afghanistan	205.47(178.04,242.79)	0.86(0.80,0.92)	36.62(23.46,54.42)	-0.39(-0.50,-0.28)	55.59(28.64,79.62)	-0.01(-0.17,0.14)

Benin	42.13(36.31,49.89)	0.55(0.53,0.57)	7.30(4.45,10.92)	-0.34(-0.39,-0.29)	54.18(37.38,73.29)	0.26(0.14,0.38)
Burkina Faso	44.26(38.48,51.48)	0.87(0.83,0.91)	7.71(4.73,11.66)	-0.16(-0.22,-0.10)	81.61(50.73,119.45)	1.08(0.89,1.26)
Burundi	46.92(41.31,54.43)	1.17(1.12,1.22)	8.36(5.22,12.55)	-0.47(-0.56,-0.38)	106.40(65.20,192.59)	-0.07(-0.26,0.13)
Central African Republic	41.42(36.64,47.13)	0.37(0.33,0.42)	11.03(7.06,16.29)	-0.07(-0.09,-0.04)	135.96(86.08,197.04)	0.34(0.23,0.46)
Chad	39.28(33.91,45.57)	0.32(0.30,0.34)	8.13(5.05,12.33)	-0.41(-0.48,-0.33)	63.73(44.17,83.44)	0.28(0.07,0.50)
C 么 te d'Ivoire	40.85(35.25,48.16)	0.51(0.49,0.53)	7.14(4.33,10.64)	-0.29(-0.35,-0.23)	46.68(32.35,62.18)	-0.02(-0.34,0.29)
Eritrea	47.05(41.64,55.29)	1.37(1.31,1.43)	8.87(5.64,13.15)	-0.54(-0.58,-0.51)	113.69(72.73,198.99)	0.89(0.74,1.03)
Ethiopia	54.72(46.23,64.89)	1.40(1.35,1.46)	6.66(4.20,9.89)	-1.06(-1.11,-1.01)	88.12(55.03,190.70)	-1.40(-1.55,-1.26)
Gambia	41.73(35.76,49.05)	0.37(0.35,0.38)	7.01(4.41,10.57)	-0.31(-0.33,-0.28)	46.74(32.88,61.93)	-0.67(-0.90,-0.44)
Guinea	38.40(33.08,45.33)	0.43(0.42,0.45)	7.51(4.63,11.47)	-0.45(-0.49,-0.40)	50.36(33.98,72.04)	0.09(-0.04,0.22)
Guinea-Bissau	38.78(33.57,44.46)	0.59(0.57,0.62)	7.98(5.00,11.94)	-0.61(-0.65,-0.56)	68.79(42.53,93.30)	-0.03(-0.19,0.13)
Haiti	282.47(245.93,328.22)	0.72(0.65,0.79)	50.70(32.50,74.89)	-0.69(-0.73,-0.66)	68.07(28.58,103.84)	-1.45(-1.63,-1.27)
Liberia	39.62(33.70,46.60)	0.75(0.70,0.79)	6.60(4.14,9.80)	-0.58(-0.67,-0.49)	49.93(26.98,78.13)	-0.26(-0.38,-0.15)
Madagascar	43.54(37.97,50.99)	0.80(0.77,0.83)	7.86(4.99,11.69)	-0.13(-0.22,-0.04)	74.47(45.86,105.26)	0.57(0.47,0.68)
Malawi	51.13(45.18,59.25)	0.87(0.80,0.93)	8.25(5.20,11.96)	-0.28(-0.35,-0.21)	87.44(59.43,125.56)	-0.25(-0.34,-0.17)
Mali	44.24(38.15,51.62)	0.96(0.91,1.01)	7.72(4.78,11.57)	-0.27(-0.36,-0.19)	59.56(40.21,86.88)	0.13(0.05,0.21)
Mozambique	52.54(46.35,60.97)	1.20(1.15,1.26)	9.16(5.86,13.32)	-0.37(-0.48,-0.26)	114.33(71.98,173.74)	1.32(1.08,1.56)
Nepal	83.16(71.15,97.86)	1.64(1.31,1.96)	11.66(7.47,17.13)	-0.07(-0.33,0.20)	22.53(13.30,41.69)	-2.78(-3.16,-2.39)

Niger	44.24(38.36,51.09)	0.97(0.94,1.00)	8.16(5.11,12.15)	-0.42(-0.47,-0.38)	66.51(42.99,95.43)	0.24(0.15,0.33)
Pakistan	162.80(137.44,191.12)	0.50(0.46,0.53)	16.62(10.52,24.65)	-0.68(-0.71,-0.65)	22.99(16.22,30.79)	-1.51(-1.55,-1.46)
Papua New Guinea	131.55(112.89,154.49)	0.37(0.34,0.39)	21.27(13.38,31.91)	-0.14(-0.17,-0.12)	55.93(34.47,102.44)	0.49(0.37,0.62)
Rwanda	57.05(50.49,66.03)	1.33(1.28,1.39)	8.59(5.42,12.87)	-1.10(-1.18,-1.02)	94.21(60.21,169.64)	-1.97(-2.27,-1.68)
Senegal	41.64(35.57,48.58)	0.58(0.56,0.61)	7.02(4.37,10.26)	-0.40(-0.45,-0.35)	43.52(26.50,61.80)	-0.09(-0.24,0.06)
Sierra Leone	42.12(36.41,48.97)	1.00(0.93,1.07)	7.54(4.75,11.22)	-0.03(-0.06,0.01)	54.40(36.09,79.73)	0.91(0.74,1.07)
Solomon Islands	173.58(151.87,203.66)	0.35(0.32,0.38)	24.64(15.81,36.50)	-0.25(-0.29,-0.22)	78.19(40.99,106.94)	0.03(-0.07,0.13)
Somalia	38.06(33.42,43.73)	0.80(0.73,0.86)	9.06(5.84,13.48)	-0.18(-0.21,-0.14)	81.09(44.14,128.86)	0.07(-0.04,0.18)
South Sudan	39.10(33.84,45.77)	0.51(0.46,0.55)	7.83(4.86,11.85)	-0.15(-0.21,-0.09)	72.17(39.64,133.44)	0.05(-0.03,0.12)
Togo	44.50(38.86,51.70)	0.63(0.56,0.70)	7.38(4.59,11.15)	-0.21(-0.24,-0.17)	54.88(36.70,74.32)	0.61(0.50,0.73)
Uganda	49.34(43.51,57.02)	0.67(0.63,0.71)	7.57(4.74,11.09)	-0.46(-0.52,-0.40)	79.65(53.31,129.53)	-0.19(-0.35,-0.04)
United Republic of Tanzania	48.60(42.46,57.00)	0.69(0.66,0.72)	7.45(4.68,11.15)	-0.27(-0.33,-0.21)	75.49(48.32,109.19)	0.34(0.14,0.53)
United States of America	853.84(727.96,1000.81)	0.49(-0.03,1.01)	48.44(31.50,70.22)	0.53(0.07,0.99)	18.97(16.31,21.63)	-0.39(-0.47,-0.32)
Yemen	229.67(198.99,267.77)	0.73(0.70,0.76)	29.09(18.69,42.92)	-0.43(-0.47,-0.39)	40.29(24.80,63.94)	0.22(0.11,0.32)

YLDs= years lived with disability; YLLs= years of life lost; UI= uncertainty intervals; CI= confidence intervals; EAPC= estimated annual percentage change;
