# Supplementary material for: The increasing incidence and high body mass index-related burden of gallbladder and biliary diseases–A results from global burden of disease study 2019
Source: Front Med (Lausanne). 2022 Dec 2;9:1002325. doi: 10.3389/fmed.2022.1002325 (PMC9757069; doi:10.3389/fmed.2022.1002325)
Supplement: Supplementary file 7 [file Table_5.pdf]

Supplementary Table 5. The high BMI contributed to age-standardized rates of YLDs and YLLs for gallbladder and biliary diseases in 2019, and estimated annual percentage change from 1990 to 2019 across 21 GBD regions

Location	Age-standardized YLD rate per 100,000 (95%UI) in 2019	EAPC of age-standardized YLD rate (95%CI)	Age-standardized YLL rate per 100,000 (95%UI) in 2019	EAPC of age-standardized YLL rate (95%CI)
Australasia	20.25(11.27,32.87)	0.80(0.64,0.95)	7.01(4.48,9.87)	-0.01(-0.08,0.06)
High-income Asia Pacific	13.23(4.80,27.07)	0.08(-0.01,0.16)	3.51(1.35,6.39)	-1.32(-1.51,-1.13)
High-income North America	24.94(14.42,40.10)	1.01(0.61,1.41)	9.05(6.02,12.11)	0.33(0.23,0.42)
Western Europe	24.86(13.13,42.74)	-0.15(-0.36,0.06)	7.86(4.80,11.33)	-0.14(-0.33,0.05)
Central Europe	36.47(20.67,59.54)	-0.30(-0.40,-0.20)	9.66(6.27,13.64)	-1.95(-2.37,-1.54)
East Asia	15.95(6.23,31.95)	1.84(1.69,2.00)	3.02(1.27,5.54)	-2.41(-2.57,-2.24)
Eastern Europe	24.76(14.21,40.67)	-0.42(-0.45,-0.39)	11.03(7.31,17.46)	-1.82(-2.04,-1.59)
Southern Latin America	6.92(3.66,11.63)	-0.65(-1.07,-0.24)	17.70(10.83,26.66)	-0.90(-1.17,-0.63)
Andean Latin America	12.57(6.95,20.51)	-0.87(-1.07,-0.68)	26.82(16.36,40.07)	-0.76(-1.08,-0.44)
Caribbean	13.71(7.34,23.03)	0.30(0.20,0.41)	11.21(6.81,16.80)	-0.88(-1.29,-0.46)
Central Asia	20.06(11.23,33.33)	0.07(0.03,0.11)	9.71(6.26,13.66)	-1.17(-1.30,-1.03)
Central Latin America	45.04(24.25,75.03)	0.62(0.48,0.75)	24.85(15.75,35.20)	0.38(0.13,0.64)
North Africa and Middle East	13.03(7.55,21.15)	0.31(0.19,0.43)	14.91(9.60,21.47)	0.01(-0.12,0.14)
Southeast Asia	7.10(3.57,12.53)	2.39(2.27,2.51)	11.40(6.18,18.15)	1.49(1.44,1.55)

Southern Sub-Saharan Africa	5.59(3.18,8.91)	0.35(0.18,0.51)	23.82(16.32,32.42)	1.12(0.75,1.48)
Tropical Latin America	37.46(21.11,61.87)	1.84(1.56,2.12)	27.24(18.21,37.16)	1.50(1.32,1.68)
Central Sub-Saharan Africa	1.66(0.75,3.19)	0.03(-0.30,0.36)	15.24(6.83,26.92)	0.17(-0.13,0.47)
Oceania	6.43(3.10,11.65)	0.00(-0.16,0.16)	14.91(7.21,27.65)	0.44(0.27,0.61)
South Asia	9.55(4.75,16.92)	4.76(4.40,5.12)	3.54(1.86,5.89)	0.80(0.66,0.94)
Eastern Sub-Saharan Africa	1.80(0.89,3.28)	1.97(1.79,2.16)	18.95(9.18,35.53)	2.39(2.21,2.58)
Western Sub-Saharan Africa	1.95(1.02,3.40)	1.79(1.74,1.83)	13.66(7.11,23.51)	2.63(2.50,2.75)

YLDs= years lived with disability; YLLs= years of life lost; UI= uncertainty intervals; CI= confidence intervals; EAPC= estimated annual percentage change; BMI= body mass index
