# Supplementary material for: The increasing incidence and high body mass index-related burden of gallbladder and biliary diseases–A results from global burden of disease study 2019
Source: Front Med (Lausanne). 2022 Dec 2;9:1002325. doi: 10.3389/fmed.2022.1002325 (PMC9757069; doi:10.3389/fmed.2022.1002325)
Supplement: Supplementary file 8 [file Table_6.pdf]

Supplementary Table 6. The high BMI contributed to age-standardized rates of YLDs and YLLs for gallbladder and biliary diseases in 2019, and estimated annual percentage change from 1990 to 2019 across 204 countries and territories

Location	Age-standardized YLDs rate per 100,000 (95%UI) in 2019	EAPC of age-standardized YLDs rate (95%CI)	Age-standardized YLLs rate per 100,000 (95%UI) in 2019	EAPC of age-standardized YLLs rate (95%CI)
Andorra	11.93(6.23,19.79)	0.19(0.13,0.26)	6.24(3.16,11.28)	0.06(-0.12,0.25)
Australia	18.34(10.21,29.90)	0.54(0.45,0.63)	7.14(4.46,10.24)	-0.10(-0.16,-0.03)
Austria	22.03(11.46,37.51)	-0.51(-0.72,-0.30)	4.45(2.59,6.65)	-1.51(-2.01,-1.00)
Belgium	11.16(5.92,19.04)	0.94(0.31,1.58)	7.74(4.51,12.06)	-0.98(-1.06,-0.90)
Bermuda	13.92(7.65,22.47)	-0.35(-0.48,-0.22)	4.40(2.59,6.87)	-3.86(-4.43,-3.28)
Brunei Darussalam	21.63(9.35,39.65)	1.73(1.62,1.84)	15.59(7.35,25.87)	1.47(1.34,1.59)
Canada	21.05(11.48,34.66)	0.46(0.38,0.53)	7.17(4.50,10.39)	-0.08(-0.16,0.01)
Cyprus	8.24(4.12,14.59)	0.78(0.10,1.46)	9.83(4.71,15.55)	-1.41(-1.59,-1.23)
Czechia	41.02(22.68,67.02)	-0.10(-0.18,-0.02)	13.45(8.41,19.83)	-1.47(-2.21,-0.72)
Denmark	15.76(8.51,27.22)	-0.37(-0.55,-0.18)	6.96(3.99,10.28)	0.07(-0.34,0.48)
Estonia	36.82(20.86,59.13)	-0.36(-0.40,-0.33)	6.47(3.94,10.38)	-3.37(-3.75,-3.00)
Finland	13.16(7.06,22.09)	-0.21(-0.92,0.51)	6.27(3.65,9.41)	-1.98(-2.15,-1.81)
France	10.18(5.36,17.41)	-0.38(-0.53,-0.23)	5.78(3.29,8.95)	-1.17(-1.29,-1.04)
Georgia	21.09(11.48,35.84)	1.10(0.82,1.39)	3.48(2.10,5.40)	-0.19(-0.60,0.22)
Germany	19.72(10.82,32.71)	-0.06(-0.61,0.50)	7.47(4.58,11.01)	-0.90(-1.62,-0.18)

Guam	7.60(4.20,12.38)	0.47(0.39,0.56)	6.57(3.86,9.83)	0.15(-0.07,0.37)
Iceland	7.99(4.26,13.54)	1.09(0.68,1.50)	4.84(2.84,7.54)	-1.54(-1.73,-1.35)
Ireland	12.67(6.72,21.31)	0.24(0.18,0.29)	6.12(3.65,9.08)	-0.50(-0.62,-0.39)
Japan	12.43(4.12,26.68)	0.15(0.08,0.22)	2.96(1.05,5.78)	-0.85(-0.97,-0.72)
Kuwait	11.15(6.50,17.70)	0.57(0.44,0.70)	6.96(4.44,10.01)	0.06(-0.26,0.37)
Latvia	46.93(26.99,76.05)	-0.16(-0.36,0.04)	8.08(4.95,12.18)	-2.32(-2.55,-2.08)
Lithuania	47.39(26.00,78.23)	0.11(-0.17,0.40)	12.18(7.12,18.47)	-0.04(-0.46,0.38)
Luxembourg	12.67(6.61,21.89)	-0.32(-0.90,0.27)	6.58(3.73,10.09)	-1.27(-1.42,-1.12)
Monaco	13.69(7.37,22.93)	0.11(0.09,0.12)	6.84(3.92,10.51)	0.39(0.20,0.57)
Netherlands	11.61(6.08,20.04)	0.37(0.32,0.43)	6.80(3.74,10.30)	-0.79(-0.90,-0.69)
New Zealand	31.18(17.25,51.24)	1.77(1.41,2.13)	6.29(3.49,9.49)	0.46(0.28,0.64)
Norway	30.74(15.64,53.16)	0.28(0.22,0.33)	5.28(2.99,7.82)	-1.45(-1.67,-1.22)
Puerto Rico	15.92(9.14,25.44)	-0.21(-0.26,-0.16)	11.89(7.37,17.49)	-1.85(-2.07,-1.62)
Qatar	8.23(4.79,12.83)	-0.78(-0.86,-0.69)	9.44(5.66,14.60)	0.79(0.46,1.12)
Republic of Korea	14.84(5.94,28.99)	-0.64(-0.81,-0.48)	6.49(2.83,11.33)	-2.75(-3.00,-2.51)
Republic of Moldova	38.42(21.64,63.70)	-0.18(-0.31,-0.04)	7.06(4.39,10.77)	-2.45(-3.00,-1.90)
San Marino	11.98(6.30,20.16)	0.21(0.20,0.22)	2.84(1.45,4.84)	0.08(-0.08,0.23)
Singapore	19.30(9.65,33.89)	1.33(1.17,1.49)	4.23(2.20,6.78)	-0.52(-0.78,-0.26)

Slovakia	50.90(28.67,83.99)	-0.53(-0.59,-0.47)	13.59(8.51,19.78)	-1.29(-1.60,-0.98)
Slovenia	35.80(20.22,58.48)	0.10(-0.07,0.28)	10.27(5.61,15.60)	-1.36(-1.62,-1.10)
Sweden	16.50(8.71,28.17)	1.28(0.94,1.62)	5.66(3.31,8.61)	-0.28(-0.47,-0.10)
Switzerland	10.27(5.28,17.73)	0.20(-0.48,0.89)	4.04(2.14,6.31)	0.34(0.17,0.50)
Taiwan (Province of China)	4.97(2.34,8.99)	0.11(-0.14,0.36)	8.33(3.81,14.24)	-1.57(-1.72,-1.42)
United Arab Emirates	10.02(5.96,15.72)	0.00(-0.10,0.09)	11.18(4.88,19.49)	-0.04(-0.76,0.68)
United Kingdom	62.39(33.85,105.02)	0.35(-0.35,1.05)	11.76(7.08,16.74)	2.17(2.01,2.32)
American Samoa	11.53(6.81,17.95)	0.17(0.10,0.25)	31.55(20.37,44.70)	1.04(0.63,1.44)
Antigua and Barbuda	14.33(7.76,23.81)	0.75(0.69,0.81)	10.30(5.99,15.54)	-0.26(-0.61,0.10)
Argentina	6.81(3.53,11.72)	-0.78(-1.35,-0.20)	17.97(10.67,27.76)	-0.23(-0.53,0.08)
Armenia	22.69(12.44,37.74)	0.94(0.82,1.05)	19.33(10.57,28.03)	1.70(1.13,2.28)
Bahamas	17.48(9.70,28.14)	-0.09(-0.15,-0.02)	18.10(10.55,27.49)	-0.89(-1.19,-0.60)
Bahrain	10.76(6.13,17.17)	-1.29(-1.39,-1.20)	15.42(9.41,22.99)	-3.33(-3.78,-2.88)
Barbados	14.88(8.00,24.28)	-0.10(-0.16,-0.05)	8.24(4.71,12.94)	-1.27(-1.54,-1.00)
Belarus	36.97(20.65,60.73)	-0.13(-0.19,-0.08)	9.14(5.22,15.02)	-1.44(-1.76,-1.11)
Bosnia and Herzegovina	38.53(20.95,63.73)	0.29(0.20,0.37)	9.16(5.58,13.92)	-0.58(-0.72,-0.45)
Bulgaria	37.52(20.49,62.12)	-0.40(-0.54,-0.27)	6.42(3.68,10.39)	-1.05(-1.72,-0.37)
Chile	7.18(3.86,11.99)	-0.65(-0.74,-0.56)	17.35(10.69,25.90)	-2.81(-3.07,-2.55)

China	16.36(6.39,32.77)	1.86(1.70,2.02)	2.91(1.20,5.36)	-2.49(-2.66,-2.32)
Cook Islands	8.96(5.16,14.42)	0.24(0.19,0.29)	29.69(17.49,44.59)	-0.94(-1.05,-0.84)
Croatia	37.48(20.80,61.80)	0.60(0.45,0.74)	9.08(5.26,13.71)	0.18(-0.20,0.55)
Dominica	15.48(8.59,25.06)	0.14(0.10,0.19)	8.82(5.42,13.01)	-1.28(-1.52,-1.04)
Greece	12.30(6.47,20.92)	0.54(0.50,0.57)	7.12(4.05,10.81)	2.02(1.52,2.52)
Greenland	27.63(15.23,45.22)	0.10(0.04,0.16)	16.43(9.68,24.62)	-1.71(-1.97,-1.46)
Hungary	46.40(26.28,76.38)	-0.57(-0.64,-0.51)	15.99(10.07,23.17)	-1.75(-2.18,-1.33)
Israel	13.23(7.11,22.63)	0.14(0.06,0.21)	10.35(6.08,15.60)	0.71(0.30,1.13)
Italy	38.68(19.17,68.80)	-0.70(-1.16,-0.25)	6.50(3.86,9.73)	-0.63(-0.84,-0.41)
Jordan	12.91(7.35,20.59)	-0.45(-0.51,-0.40)	15.05(9.44,21.71)	-1.77(-1.92,-1.61)
Kazakhstan	22.26(12.38,35.76)	-0.49(-0.64,-0.34)	11.44(7.30,16.65)	-2.63(-3.20,-2.06)
Lebanon	11.26(6.31,18.21)	0.34(0.21,0.46)	8.12(3.63,14.85)	0.83(0.60,1.06)
Libya	12.00(6.86,18.88)	0.14(0.09,0.18)	9.95(5.45,15.65)	0.31(0.14,0.48)
Malaysia	9.82(5.25,16.51)	0.97(0.89,1.06)	21.16(12.26,32.36)	1.71(1.44,1.98)
Malta	5.35(2.69,9.27)	1.16(0.67,1.66)	5.29(3.03,8.20)	-0.84(-1.01,-0.66)
Mauritius	8.19(4.37,13.99)	1.06(1.01,1.10)	5.68(3.12,8.98)	2.23(1.82,2.64)
Montenegro	37.96(20.87,62.16)	0.15(0.06,0.23)	3.30(2.14,4.62)	0.22(0.08,0.36)
Niue	8.76(4.82,14.50)	0.23(0.17,0.29)	26.10(14.97,41.07)	-0.11(-0.22,0.00)

North Macedonia	36.29(19.52,61.40)	0.03(-0.01,0.07)	4.65(2.89,6.94)	-1.14(-1.28,-1.00)
Northern Mariana Islands	10.51(5.91,17.03)	0.02(-0.09,0.13)	34.10(20.86,51.09)	-0.73(-0.93,-0.54)
Oman	9.36(5.34,15.15)	0.95(0.73,1.17)	5.24(2.61,8.15)	1.37(1.12,1.61)
Palau	8.73(4.90,14.34)	-0.10(-0.14,-0.06)	24.31(12.64,39.74)	0.13(0.08,0.18)
Panama	34.61(18.69,59.01)	1.51(1.43,1.59)	8.78(4.74,14.38)	-0.16(-0.39,0.07)
Poland	23.45(13.21,38.28)	-1.04(-1.52,-0.56)	8.85(5.59,13.69)	-3.11(-3.52,-2.69)
Portugal	5.93(2.95,10.26)	1.35(1.16,1.55)	8.14(4.45,12.59)	0.79(0.62,0.97)
Romania	49.73(28.95,78.37)	0.24(0.11,0.37)	6.63(4.09,11.55)	-2.43(-2.92,-1.93)
Russian Federation	21.04(11.91,33.88)	-0.28(-0.30,-0.26)	12.69(8.29,19.86)	-1.72(-1.95,-1.48)
Saint Kitts and Nevis	15.87(8.66,25.78)	-0.60(-0.72,-0.48)	18.25(10.28,30.35)	-2.74(-3.24,-2.24)
Saudi Arabia	12.36(7.26,19.61)	-0.09(-0.22,0.05)	18.87(12.08,27.58)	-1.50(-1.74,-1.25)
Serbia	37.36(20.61,61.41)	0.30(0.21,0.38)	11.34(7.16,16.67)	0.36(0.19,0.52)
Seychelles	8.70(4.60,15.02)	-0.15(-0.25,-0.06)	5.27(2.42,9.25)	0.01(-0.15,0.17)
Spain	9.09(4.89,15.48)	-0.59(-0.73,-0.45)	11.10(6.48,16.25)	-0.07(-0.28,0.13)
Sri Lanka	6.41(3.04,11.31)	1.38(1.27,1.48)	1.73(0.85,2.87)	0.84(0.59,1.08)
Thailand	7.79(3.99,13.99)	0.39(0.24,0.55)	14.12(7.43,23.09)	0.44(0.28,0.59)
Trinidad and Tobago	16.80(9.51,26.48)	-0.02(-0.09,0.06)	12.68(7.17,20.14)	-1.28(-1.53,-1.02)
Turkey	13.03(7.49,21.37)	0.57(0.24,0.91)	14.09(8.65,20.35)	-0.80(-1.03,-0.57)

Ukraine	30.03(17.16,49.14)	-0.66(-0.73,-0.60)	6.89(4.23,12.11)	-2.72(-3.17,-2.27)
United States Virgin Islands	19.36(11.02,30.93)	0.01(-0.04,0.07)	22.10(14.17,31.67)	-0.53(-0.73,-0.33)
Uruguay	6.82(3.62,11.42)	0.85(0.76,0.95)	16.84(9.76,25.12)	0.83(0.58,1.08)
Albania	32.93(17.32,57.49)	0.83(0.71,0.94)	3.29(1.85,5.27)	-0.65(-0.94,-0.36)
Algeria	11.70(6.53,19.06)	0.11(0.10,0.13)	8.96(5.25,13.41)	0.13(0.01,0.25)
Azerbaijan	20.25(11.19,33.54)	0.14(-0.03,0.30)	7.79(3.98,17.36)	-1.62(-2.03,-1.21)
Botswana	6.76(3.97,10.86)	2.36(1.13,3.59)	39.19(22.00,65.35)	1.91(1.43,2.39)
Brazil	37.67(21.25,62.28)	1.87(1.58,2.16)	27.42(18.36,37.63)	1.51(1.32,1.69)
Colombia	38.28(19.94,65.39)	0.74(0.67,0.80)	18.69(10.92,29.08)	0.77(0.55,0.99)
Costa Rica	37.88(19.95,63.41)	0.34(0.30,0.38)	15.16(8.75,24.30)	-0.25(-0.62,0.13)
Cuba	14.01(7.66,23.39)	0.11(-0.02,0.25)	10.67(6.39,16.62)	-1.44(-1.98,-0.90)
Ecuador	15.01(8.52,24.01)	-0.30(-0.44,-0.16)	35.08(21.74,51.45)	0.59(-0.12,1.30)
Egypt	17.22(10.02,28.02)	-0.21(-0.26,-0.17)	36.67(19.99,60.47)	0.16(0.05,0.26)
Equatorial Guinea	3.00(1.64,4.95)	4.58(3.87,5.29)	28.69(14.86,48.78)	5.09(4.39,5.79)
Fiji	9.95(5.56,16.06)	0.38(0.31,0.46)	18.21(10.56,28.32)	1.93(1.65,2.21)
Gabon	2.84(1.56,4.68)	1.18(0.97,1.38)	26.27(12.47,50.05)	1.75(1.23,2.27)
Grenada	15.39(8.37,25.61)	0.65(0.58,0.73)	13.21(7.92,20.03)	-0.33(-0.68,0.02)
Guyana	17.91(9.71,29.94)	0.68(0.63,0.73)	20.46(11.45,32.49)	-0.04(-0.40,0.32)

Indonesia	6.98(3.49,12.24)	3.57(3.36,3.78)	14.57(6.89,24.60)	1.90(1.78,2.01)
Iran (Islamic Republic of)	14.49(7.98,23.97)	0.85(0.42,1.29)	8.20(4.61,11.47)	1.64(1.31,1.98)
Iraq	11.22(6.24,18.64)	-0.62(-0.66,-0.57)	5.65(3.40,8.50)	-1.89(-2.04,-1.74)
Jamaica	15.21(8.58,24.91)	0.50(0.44,0.55)	7.89(4.75,12.05)	0.27(-0.27,0.82)
Mexico	51.39(27.76,84.72)	0.71(0.49,0.93)	30.98(19.55,44.14)	0.46(0.15,0.77)
Namibia	6.12(3.47,9.97)	0.21(0.13,0.30)	31.36(16.75,54.90)	0.51(0.26,0.76)
Nauru	9.93(5.35,16.64)	0.08(0.00,0.16)	35.99(17.92,60.65)	-0.35(-0.48,-0.22)
Paraguay	30.19(16.32,50.86)	0.51(0.47,0.55)	19.63(11.16,31.21)	0.98(0.66,1.31)
Peru	10.86(5.67,18.19)	-1.48(-1.82,-1.14)	19.07(10.14,31.12)	-1.86(-2.19,-1.54)
Philippines	9.55(4.42,16.88)	2.30(2.19,2.41)	9.16(4.88,14.92)	0.55(0.31,0.79)
Saint Lucia	14.41(7.99,23.80)	0.26(0.19,0.32)	7.84(4.46,12.34)	-1.50(-1.97,-1.03)
Saint Vincent and the Grenadines	14.95(8.22,25.08)	0.99(0.95,1.04)	12.23(7.28,18.44)	0.14(-0.27,0.54)
Samoa	10.24(5.68,16.80)	-0.34(-0.41,-0.28)	27.25(14.77,43.86)	-0.37(-0.41,-0.33)
South Africa	5.41(3.08,8.64)	0.15(0.01,0.29)	22.48(14.51,30.60)	0.80(0.39,1.21)
Suriname	17.20(9.08,28.52)	0.36(0.29,0.43)	17.79(10.62,26.79)	-0.82(-1.22,-0.42)
Syrian Arab Republic	11.04(6.11,17.92)	-0.07(-0.15,0.01)	6.39(3.05,10.39)	-1.23(-1.50,-0.96)
Tokelau	8.83(4.65,14.62)	0.16(0.04,0.29)	23.80(13.07,38.90)	0.03(-0.07,0.14)
Tonga	11.42(6.41,18.27)	-0.21(-0.33,-0.10)	31.25(17.59,48.92)	-0.24(-0.42,-0.05)

Tunisia	7.03(3.81,11.44)	-0.47(-0.81,-0.13)	8.28(4.31,13.89)	1.57(1.41,1.73)
Turkmenistan	20.75(11.50,34.13)	0.26(0.23,0.28)	9.52(5.28,15.21)	-0.61(-0.86,-0.35)
Uzbekistan	20.01(10.95,33.47)	0.51(0.46,0.56)	9.92(6.02,14.60)	0.80(0.51,1.08)
Viet Nam	4.10(1.59,8.32)	3.06(2.81,3.31)	5.51(2.31,10.21)	1.42(1.23,1.62)
Angola	1.81(0.81,3.35)	3.47(3.25,3.68)	16.82(6.72,32.88)	4.00(3.71,4.30)
Bangladesh	3.28(1.35,6.36)	4.78(4.48,5.08)	2.91(1.16,5.97)	2.07(1.74,2.39)
Belize	19.00(10.46,30.63)	0.66(0.50,0.81)	18.99(11.68,27.54)	0.48(0.24,0.73)
Bhutan	5.06(2.44,9.14)	1.60(1.41,1.79)	4.58(1.71,11.04)	0.79(0.71,0.88)
Bolivia (Plurinational State of)	14.32(7.83,23.60)	-0.06(-0.12,-0.01)	41.34(22.62,69.90)	-0.21(-0.28,-0.14)
Cabo Verde	1.87(1.00,3.21)	1.32(1.19,1.44)	7.88(4.69,12.27)	1.42(1.22,1.62)
Cambodia	5.60(2.27,11.49)	1.83(1.67,1.98)	9.16(3.70,18.12)	1.69(1.62,1.75)
Cameroon	2.79(1.51,4.59)	0.10(0.03,0.17)	17.68(9.52,29.32)	0.40(0.23,0.57)
Comoros	2.33(1.16,4.15)	0.70(0.64,0.76)	24.12(10.83,46.82)	1.08(0.90,1.26)
Congo	2.68(1.43,4.54)	1.25(1.19,1.30)	28.59(14.48,48.41)	1.88(1.57,2.20)
Democratic People's Republic of Korea	3.29(0.60,8.61)	-0.54(-0.59,-0.49)	2.61(0.50,6.52)	-1.52(-1.58,-1.46)
Democratic Republic of the Congo	1.49(0.61,2.93)	-1.04(-1.47,-0.60)	13.02(5.52,24.29)	-1.12(-1.58,-0.65)
Djibouti	1.61(0.75,3.06)	3.21(3.02,3.40)	13.77(5.24,28.12)	3.67(3.45,3.90)
Dominican Republic	13.38(7.05,22.49)	1.69(1.43,1.96)	7.91(4.24,13.48)	1.35(0.60,2.10)

El Salvador	44.48(23.51,74.46)	0.12(0.09,0.16)	17.62(10.27,27.41)	-1.37(-2.00,-0.74)
Eswatini	7.67(4.41,12.30)	0.09(-0.09,0.28)	42.65(23.59,70.69)	1.41(0.78,2.04)
Ghana	2.65(1.43,4.40)	2.84(2.58,3.10)	20.24(11.85,32.28)	4.39(4.11,4.66)
Guatemala	38.59(18.98,69.08)	0.95(0.86,1.05)	17.42(9.34,28.29)	0.14(-0.65,0.93)
Honduras	49.08(24.58,85.85)	1.16(0.95,1.36)	60.39(32.85,103.51)	2.01(1.62,2.41)
India	10.98(5.48,19.28)	4.93(4.54,5.33)	3.34(1.72,5.67)	0.66(0.46,0.85)
Kenya	2.14(1.11,3.73)	1.38(1.24,1.53)	27.46(12.39,52.27)	2.70(2.51,2.88)
Kiribati	12.57(6.44,21.55)	-0.25(-0.42,-0.08)	43.50(19.01,76.93)	-0.87(-1.08,-0.66)
Kyrgyzstan	17.17(9.00,28.64)	-0.53(-0.61,-0.46)	7.09(4.04,12.05)	-2.46(-2.78,-2.13)
Lao People's Democratic Republic	8.45(3.84,15.19)	3.02(2.90,3.13)	11.86(4.54,23.45)	2.16(2.08,2.24)
Lesotho	7.85(4.38,12.76)	1.62(1.36,1.88)	43.19(23.43,71.35)	3.82(3.33,4.32)
Maldives	5.29(2.49,9.56)	2.93(2.67,3.18)	1.31(0.61,2.31)	-0.59(-0.80,-0.37)
Marshall Islands	8.73(4.13,15.81)	0.40(0.20,0.60)	26.86(11.18,52.68)	0.19(-0.05,0.44)
Mauritania	2.45(1.32,4.15)	0.25(0.17,0.34)	12.98(7.42,20.84)	-0.28(-0.43,-0.14)
Micronesia (Federated States of)	11.16(6.00,18.12)	-0.40(-0.54,-0.25)	34.72(16.13,62.26)	-0.57(-0.62,-0.52)
Mongolia	20.68(10.52,35.62)	-0.82(-0.90,-0.73)	16.46(8.92,27.48)	-3.06(-3.42,-2.71)
Morocco	12.13(6.56,20.11)	0.58(0.55,0.61)	11.14(6.45,17.24)	1.30(1.19,1.41)
Myanmar	6.92(3.26,12.74)	3.71(3.48,3.93)	5.07(2.27,9.44)	2.78(2.50,3.07)

Nicaragua	36.76(18.82,62.05)	0.43(0.33,0.52)	16.51(9.75,28.02)	0.47(0.23,0.72)
Nigeria	1.68(0.86,2.95)	2.16(2.11,2.20)	13.47(5.21,30.58)	2.88(2.68,3.09)
Palestine	11.21(6.10,18.38)	-0.10(-0.23,0.04)	9.56(4.33,15.87)	0.07(-0.18,0.31)
Sao Tome and Principe	2.16(1.12,3.64)	0.89(0.85,0.94)	8.80(4.69,16.92)	0.25(-0.25,0.75)
Sudan	11.66(6.35,19.38)	1.39(1.32,1.46)	11.42(4.73,22.22)	2.74(2.54,2.94)
Tajikistan	12.79(6.06,23.82)	0.61(0.22,1.00)	5.21(2.55,8.98)	-0.34(-0.77,0.09)
Timor-Leste	3.97(1.36,8.65)	1.59(1.14,2.05)	5.37(1.45,13.37)	1.50(0.95,2.05)
Tuvalu	8.56(4.31,14.97)	0.13(-0.06,0.33)	26.16(12.34,46.58)	-0.02(-0.17,0.13)
Vanuatu	10.75(5.48,18.77)	0.47(0.34,0.60)	28.98(11.96,50.46)	0.34(0.14,0.54)
Venezuela (Bolivarian Republic of)	36.77(19.16,63.61)	-0.17(-0.34,0.00)	13.33(7.43,21.43)	-1.04(-1.53,-0.55)
Zambia	2.16(1.08,3.83)	0.79(0.54,1.04)	22.31(10.64,38.95)	1.21(1.04,1.39)
Zimbabwe	5.77(3.01,9.91)	0.75(0.61,0.89)	22.32(10.03,43.54)	2.36(2.02,2.69)
Afghanistan	13.53(7.11,23.27)	1.41(0.90,1.91)	19.14(8.48,32.05)	2.21(1.67,2.74)
Benin	2.53(1.32,4.36)	1.65(1.45,1.85)	14.39(7.60,23.38)	2.63(2.37,2.88)
Burkina Faso	2.00(0.93,3.64)	3.04(2.92,3.16)	15.83(6.61,33.33)	4.25(4.00,4.50)
Burundi	1.23(0.48,2.54)	0.60(0.43,0.77)	14.11(4.92,31.68)	0.98(0.71,1.24)
Central African Republic	1.61(0.62,3.37)	1.46(1.33,1.59)	18.16(6.76,38.73)	2.15(1.87,2.44)
Chad	1.45(0.63,2.76)	1.92(1.79,2.05)	8.95(3.78,16.31)	2.63(2.41,2.84)

C 么 te d'Ivoire	2.21(1.13,3.78)	0.88(0.79,0.98)	11.16(5.73,18.74)	1.27(1.00,1.54)
Eritrea	1.61(0.71,3.04)	2.32(1.98,2.66)	23.65(9.70,50.41)	4.23(3.77,4.69)
Ethiopia	1.18(0.50,2.29)	2.06(1.77,2.34)	13.83(5.16,37.41)	1.47(1.04,1.90)
Gambia	2.11(1.07,3.72)	1.65(1.55,1.76)	12.20(6.12,20.32)	1.63(1.41,1.85)
Guinea	1.89(0.93,3.35)	0.93(0.88,0.99)	9.84(4.55,17.39)	1.58(1.46,1.69)
Guinea-Bissau	1.80(0.81,3.34)	0.88(0.72,1.04)	13.32(5.79,24.17)	1.89(1.69,2.09)
Haiti	10.75(4.65,20.75)	0.58(0.50,0.66)	13.47(4.34,27.56)	-0.13(-0.33,0.08)
Liberia	2.41(1.29,4.13)	1.29(0.84,1.75)	13.53(6.67,24.20)	1.98(1.54,2.42)
Madagascar	1.58(0.73,2.96)	2.11(1.68,2.53)	15.29(6.47,27.86)	3.15(2.77,3.53)
Malawi	2.11(0.99,3.92)	2.63(2.47,2.78)	20.50(9.21,37.68)	3.12(2.89,3.36)
Mali	1.80(0.83,3.32)	2.78(2.66,2.90)	11.70(5.50,21.40)	2.87(2.71,3.03)
Mozambique	2.30(1.10,4.17)	3.47(3.31,3.62)	23.09(9.53,46.36)	5.10(4.75,5.46)
Nepal	2.61(1.17,4.89)	5.10(4.64,5.57)	4.53(1.72,10.04)	1.79(1.47,2.12)
Niger	1.59(0.71,3.04)	1.07(1.00,1.13)	10.41(4.40,19.82)	2.14(2.05,2.24)
Pakistan	4.45(2.13,8.15)	2.35(2.16,2.54)	6.16(2.97,10.52)	1.59(1.36,1.81)
Papua New Guinea	5.17(2.14,10.06)	0.15(-0.04,0.35)	11.37(4.07,25.62)	0.70(0.42,0.97)
Rwanda	2.08(0.94,3.85)	1.47(1.18,1.76)	20.32(8.40,42.06)	0.56(0.10,1.03)
Senegal	2.12(1.06,3.63)	0.60(0.51,0.70)	10.84(5.02,19.27)	1.61(1.43,1.79)

Sierra Leone	1.65(0.74,3.16)	1.72(1.55,1.89)	9.33(3.73,18.43)	3.18(2.96,3.41)
Solomon Islands	8.77(4.31,15.44)	0.48(0.22,0.75)	23.17(9.11,41.14)	0.72(0.39,1.06)
Somalia	0.90(0.25,2.21)	0.58(0.47,0.70)	7.10(1.40,19.12)	0.84(0.68,1.00)
South Sudan	2.40(1.21,4.27)	1.53(1.45,1.60)	19.78(8.46,38.80)	1.99(1.85,2.14)
Togo	2.12(1.08,3.77)	1.78(1.70,1.86)	13.39(6.72,22.55)	2.81(2.69,2.93)
Uganda	1.97(0.96,3.63)	3.23(3.02,3.45)	18.46(8.24,36.36)	3.55(3.38,3.71)
United Republic of Tanzania	2.40(1.22,4.14)	1.77(1.51,2.02)	21.63(10.44,38.59)	2.16(1.97,2.35)
United States of America	25.37(14.73,40.69)	1.06(0.62,1.50)	9.27(6.14,12.36)	0.36(0.26,0.47)
Yemen	8.80(4.36,15.60)	1.96(1.77,2.15)	9.78(4.30,18.10)	2.42(2.19,2.65)

YLDs= years lived with disability; YLLs= years of life lost; UI= uncertainty intervals; CI= confidence intervals; EAPC= estimated annual percentage change; BMI= body mass index
