# Supplementary material for: The increasing incidence and high body mass index-related burden of gallbladder and biliary diseases–A results from global burden of disease study 2019
Source: Front Med (Lausanne). 2022 Dec 2;9:1002325. doi: 10.3389/fmed.2022.1002325 (PMC9757069; doi:10.3389/fmed.2022.1002325)
Supplement: Supplementary file 9 [file Table_7.pdf]

Supplementary Table 7. The gallbladder and biliary diseases burden due to high BMI in 2019 by sex and estimated annual percentage change from 1990 to 2019 across global and in different SDI regions

Location	Female				Male			
	Age-standardized PAF (95%UI) in 2019	EAPC of age-standardized PAF (95%CI)	ASR per 100,000 (96%UI) in 2019	EAPC of ASR (95%CI)	Age-standardized PAF (95%UI) in 2019	EAPC of age-standardized PAF (95%CI)	ASR per 100,000 (96%UI) in 2019	EAPC of ASR (95%CI)
**YLDs**								
Global	0.37(0.24,0.51)	1.04(0.99,1.09)	24.90(13.20,43.36)	0.53(0.47,0.58)	0.23(0.12,0.38)	1.73(1.68,1.78)	7.00(3.11,13.27)	0.95(0.86,1.03)
High SDI	0.45(0.32,0.60)	0.70(0.64,0.76)	32.92(18.04,55.07)	0.30(0.21,0.39)	0.28(0.16,0.44)	1.12(1.04,1.20)	11.62(5.43,21.14)	0.83(0.73,0.93)
High-middle SDI	0.39(0.25,0.54)	0.60(0.58,0.62)	28.82(15.17,49.66)	-0.21(-0.29,-0.13)	0.24(0.12,0.39)	1.54(1.51,1.57)	9.20(4.05,17.48)	0.64(0.52,0.76)
Middle SDI	0.36(0.23,0.50)	2.02(1.92,2.13)	25.84(13.52,45.57)	1.51(1.41,1.62)	0.21(0.10,0.35)	2.87(2.79,2.95)	6.33(2.65,12.28)	1.92(1.80,2.04)
Low-middle SDI	0.29(0.17,0.42)	2.50(2.42,2.58)	19.97(9.89,36.46)	2.57(2.45,2.69)	0.17(0.08,0.29)	2.88(2.86,2.91)	3.22(1.28,6.51)	2.35(2.25,2.45)
Low SDI	0.23(0.13,0.35)	1.93(1.83,2.04)	6.98(3.28,12.90)	2.42(2.21,2.63)	0.13(0.06,0.23)	2.21(2.13,2.28)	0.96(0.36,1.99)	2.24(2.09,2.40)
**YLLs**								
Global	0.37(0.24,0.50)	1.45(1.41,1.49)	10.90(6.91,15.38)	-0.46(-0.57,-0.34)	0.23(0.12,0.37)	1.82(1.77,1.87)	6.41(3.44,10.64)	0.39(0.29,0.49)
High SDI	0.45(0.31,0.60)	0.59(0.53,0.64)	8.00(5.22,11.01)	-0.53(-0.69,-0.37)	0.29(0.16,0.45)	1.00(0.93,1.07)	6.56(3.60,10.45)	0.05(-0.06,0.16)
High-middle SDI	0.47(0.32,0.62)	1.21(1.16,1.27)	9.94(6.48,13.73)	-1.83(-1.94,-1.72)	0.28(0.16,0.44)	1.65(1.59,1.71)	6.72(3.66,10.89)	-0.55(-0.62,-0.47)
Middle SDI	0.39(0.25,0.53)	2.56(2.48,2.64)	12.14(7.70,17.28)	-0.15(-0.21,-0.08)	0.23(0.12,0.37)	2.71(2.64,2.77)	6.41(3.40,10.58)	1.11(1.02,1.20)

Low-middle SDI	0.31(0.20,0.44)	2.86(2.77,2.94)	10.57(6.37,15.69)	1.15(1.07,1.23)	0.18(0.09,0.31)	3.56(3.48,3.63)	4.73(2.30,8.30)	1.82(1.70,1.94)
Low SDI	0.24(0.14,0.37)	2.30(2.20,2.41)	14.02(7.33,23.29)	1.45(1.34,1.57)	0.13(0.06,0.24)	2.22(2.07,2.37)	6.32(2.45,12.64)	1.73(1.56,1.90)

ASR=age-standardized rate； SDI= sociodemographic index; YLDs= years lived with disability; YLLs= years of life lost; UI= uncertainty intervals; CI= confidence intervals; EAPC= estimated annual percentage change; PAF= population attributable fraction; BMI= body mass index
